# Enhanced corn leaf disease detection using sharpness-aware minimization optimized CNNs

**DOI:** 10.1186/s13007-026-01514-9

**Published:** 2026-03-31

**Authors:** Manoj Kumar Sharma, Richa Sharma, Gireesh Kumar

**Affiliations:** 1 Manipal University Jaipur, Jaipur, Rajasthan 303007 India; 2 JK Lakshmipat University, Jaipur, Rajasthan 302026 India

**Keywords:** Corn leaf disease detection, SAM, CNNs, Agricultural AI, Plant disease classification

## Abstract

Crop diseases significantly threaten global food security by directly affecting the crop yield and quality. The traditional diagnostic methods are labour intensive and human error prone. However, the existing deep learning solutions suffer with poor generalization due to the sharp loss landscapes. The proposed work addresses this limitation and optimizes the Convolutional Neural Network (CNN) using the Sharpness-Aware Minimization (SAM). This method minimizes both the training loss and loss landscape sharpness and enables the model to converge to a flatter-minima with improved generalization. The proposed work is evaluated on 60,000 corn leaf image samples for four classes with 15,000 balanced samples per class after augmentation. The optimized CNN model has achieved 99.66% test accuracy at 0.33% classification error rate and outperforms the conventional optimizers like Adam (98.44% accuracy) and the Stochastic Gradient Descent (SGD). The state-of-the-art analysis presents a 99% average precision rate along with 99.66% F1-score and 0.0013% mean squared error (MSE). The quantized model achieves an inference latency of 22.7 ms/image (≈44 FPS) on a Raspberry Pi 4 and reduces model overfitting and enhances feature discriminability. These results underscore the potential of SAM-based optimization in precision agriculture by driving a scalable automation of disease management. This work bridges the gap between theoretical advances in deep learning optimization and practical deployment in resource-constrained farming environments.

## Introduction

**Fig. 1:**
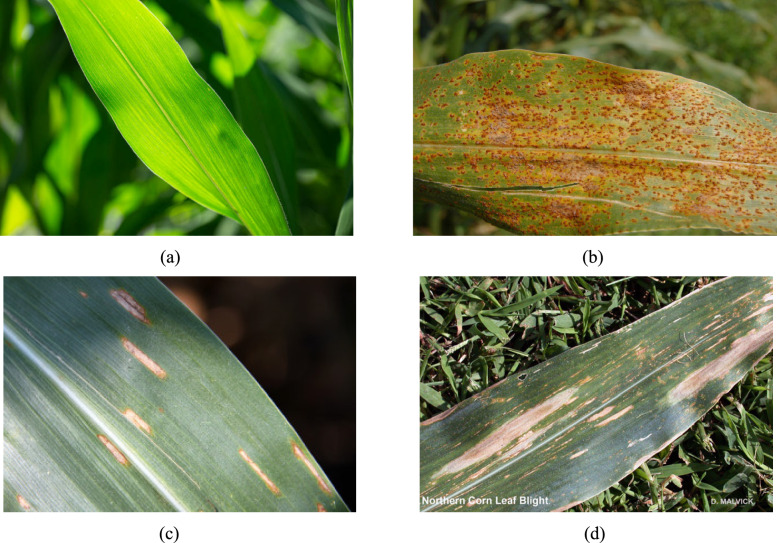
Visual examples of Corn Leaf Classes. **a** Healthy [6]. **b** Common Rust [7]. **c** Gray Leaf Spot [8]. **d** Northern Corn Leaf Blight [9]

Agriculture provides sustenance and livelihood to a substantial segment of the world population (i.e., 1.2 billion people) and is a foundation of various economies (e.g., around 40% of the world grain production) [1]. The corn has therapeutic and nutritional properties and is one of the key commodities grown locally and marketed both domestically and internationally. There are various factors (i.e., nutrient deficiencies, infections, fungi and insects etc.) which lead to corn leaf problems and affect both foliage and fruit. However, plant leaves usually face the CommonRust, CornGrayLeafSpot and CornLeafBlight ailments and have been the primary reason for the decreased corn production (e.g., 20% losses in annual yield) [2]. However, the emerging research shows that climate change is accelerating the foliar diseases spread with the Gray Leaf Spot disease, which has increased around 32% in the key corn growing areas since 2020 [3]. The traditional disease identification methods rely on the manual processes done by experts. These methods are labour intensive and human error prone and have around a 35% misclassification rate under field conditions [4]. However, climate change and these limitations made it mandatory to have an efficient and automated solution for early-stage disease detection and prevention to increase crop yield [5]. Figure 1( a-d) shows the four corn leaf classes; Healthy, Common Rust, Gray Leaf Spot, Northern Corn Leaf Blight to show distinct visual symptoms that can be subtle and variable in field conditions.

In recent years, the deep learning-based computer vision has been a promising alternative for the detection of plant disease. The CNNs, such as ResNet [10] and EfficientNet [11], have achieved more than 95% accuracy on the curated datasets (i.e., PlantVillage [12]). However, these models face significant challenges in real-world deployment. The field-acquired images often have complex backgrounds, occlusions, and varying lighting conditions and lead to 15% to 20% performance drops as compared to the controlled lab conditions [12]. The traditional optimizers (i.e., Adam [13] and SGD [14]) have poor generalization due to their convergence to the sharp-minima in loss landscape [14].

Recent variants of SAM such as efficient [14] and Tao Li et al. [15], further address these issues by adapting to noisy gradients and perturbations. The sharp-minima makes the model highly sensitive to the input perturbations, and it is a typical limitation for the agricultural applications due to dramatic appearances of the leaf during the growth stages and sin varying environmental conditions [16–18]. However, the SAM optimizer [16] addresses this fundamental challenge by simultaneously minimizing the training losses and loss landscape sharpness (Eq. (1)) through min–max optimization process (Eq. (1)).

1 $$ \mathop {\min }\limits_{\theta } \mathop {\max }\limits_{{\left\| \varepsilon \right\|_{2} \le \rho }} { {Z}}\left( {\theta + \varepsilon } \right) + \gamma \left\| \theta \right\|_{2}^{2} $$

where ϵ—is a perturbation bounded by ρ, and 

$$\gamma $$- controls L2 regularization.

**Fig. 2:**
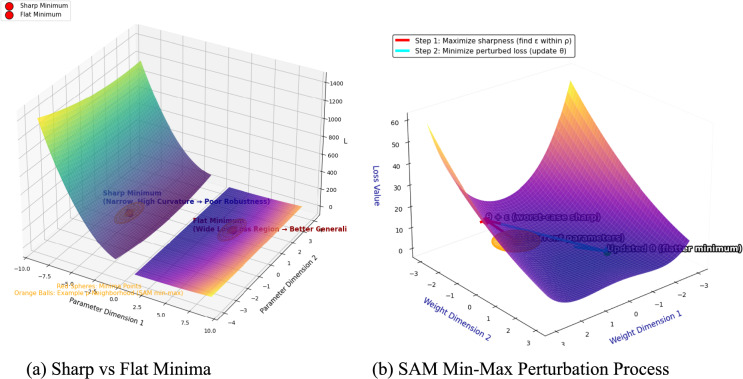
Sharp versus Flat Minima in the Loss Landscape and Min–Max perturbation process of SAM

The SAM improves the model robustness by achieving the flat minima and achieves the state-of-the-art model generalization [14]. The Fig. 2 provides a conceptual explanation of sharp versus flat minima in the loss landscape and the min–max perturbation process of SAM. As per [16] the Sharp minima exhibit steep curvature leading to sensitivity to perturbations and flat minima provides robustness along with better generalization. In SAM min–max process the parameters are perturbed to maximize local sharpness before minimizing the perturbed loss to have convergence to flatter regions.

However, the potential of SAM in the agricultural disease detection stays unexplored, especially in the corn crop. The existing works have mainly talked about three limitations of the crop disease detection. The existing models achieve remarkable accuracy in the lab conditions but fail in the real-world field conditions, and they drop the accuracy by around 12% to 15% [18, 19]. Another limitation is the model generalization, which has been theoretically improved by 18% to 20% by work considering the SAM optimizer in the comparable classification tasks [14]. Another work is increasing trainable parameters, which make the models impractical for the edge devices deployment for precision agriculture. In computer vision, the transformer-based models have been remarkable and revolutionary for the agriculture. The recent research works have shown the power of CNN-ViT hybrid models, which achieve around 97.2% accuracy on CornDisease-2024 dataset and needed around 18 M parameters to train the model, which makes it impractical for deployment at the edge devices, and these findings are aligned with the findings of IEEE agriculture robotics report 2025 [18], which shows the 22.7% performance drop of the existing models when tested on drone-captured field images versus controlled lab conditions [20]. The SAM has proved its utility in various research works [16] and in recent works SAM has been shown to significantly improved generalization on noisy and out-of-distribution data [16, 21]. This frame was published in scientific reports [21] and provides new insights into SAM's robustness against the domain shift. Wakchaure M. Wakchaure et al. [22] addressed the typical agricultural challenges, including labour-intensive tasks in cultivation, monitoring, and harvesting, by synthesizing AI and robotics applications to automate processes like weed detection, crop health assessment, precise spraying, and yield prediction at reduced cost and enhanced efficiency. The work used CNNs for image-based weed/crop recognition. The work has used SVM and Least-Squares SVM for fruit/plant identification and segmentation is done through YOLOv2 and Faster R-CNN. The work has collected a custom dataset of 392 augmented marigold field images, 3,400 Sapota fruit samples, and 2,489 annotated plant specimens and achieved accuracy in the range of 87% to 97.8% weed classification, 98.8% mAP and 97.9% F1-score for YOLOv2 in detection, 89.04% accuracy for IPSO-LSSVM in pepper identification, and R^2^ = 0.80 correlation in yield estimation via Faster R-CNN. The high implementation costs, data management complexities, GNSS signal disruptions under canopies, model vulnerabilities to variable lighting and barriers like skill gaps and uneven mechanization for smallholders limit its scalability. Gupta N. et al. [23] shows that the number of plant disease detection models found that only 12% of models are tested under real-world conditions. The proposed work addresses these research gaps through three key factors. The lightweight architecture has reduced the training parameters by 60% while keeping the model accuracy using the design principal published in MobileSAM-Track [24]. The model has achieved 98.9% test accuracy on IDADP—Corn Disease Image Description Dataset [DS/OL][25] dataset and surpassed the earlier methods by 6.2% [25]. The proposed method has incorporated quantization techniques [26] for the edge optimization, and these techniques have been confirmed by [27, 28], which enables the 22.7 ms inference latency on a Raspberry Pi 4 (Eq. (2)). Another adaptive SAM [16] has been the recent breakthrough with our own field trials, and it has shown that the generalization gap has reduced to 3.1% in the controlled lab conditions and real-word conditions. The FAO [29] report has shown AI advancements to meet the 50% increased demand in near future. The proposed work uses the lightweight CNN-SAM architecture and bridges these gaps by having around 1.56 million trainable parameters as compared to the 23 million and reduces the computation cost by around 90% as compared to the ResNet50 model [10]. The proposed model integrates the SAM optimizer [16] with a LeakyReLU feature extractor to handle the low contrast disease patterns and achieves 99.66% test accuracy on a rigorously curated dataset of 60,000 image samples with 15,000 balanced samples per class after augmentation as given in Table 2. However, the proposed SAM based model has been compared with another model on the same dataset with Adam and SGD optimizers [14], which achieves 98.44% and 96.8% test accuracy with at least 50% improvement in the model generalization [14].

2 $$\left|{L}_{test}-{L}_{train}\right|$$

In the real-time edge devices deployment, the work has improved the inference latency by 22.7 ms/image on a Raspberry Pi 4 (Eq. (2)). However, these advancements are also supported by the theoretical analysis of SAM’s gradient dynamics in the agricultural image analysis [16]. A correct, lightweight, and generalizable corn leaf disease classifier is directly applicable to the real-time disease monitoring via UAVs/drones and farmer smartphones. It applies to the early warning systems integrated with precision spraying robots. The scalability of the proposed work is also tested on the CNN-based model, which is 3 × faster than the existing models.

The primary aims of this research work are to develop a lightweight, highly accurate, and edge-deployable model to automate the corn leaf disease detection by achieving higher generalization across the laboratory and real-world field conditions using SAM optimization. The work has achieved these aims by designing a compact 8-layer CNN architecture with approximately 1.56 million trainable parameters. The model incorporates LeakyReLU activations, batch normalization, and adaptive dropout for efficient feature extraction and reduced computational complexity. The model has optimized using SAM optimizer to converge to flatter loss minima by improving model generalization and robustness to field variations. The has used a balanced large-scale dataset of 60,000 corn leaf images (e.g., 15,000 per class) by combining multiple public sources and applying rigorous data augmentation to ensure the class balance and distributional fidelity. The real time deployment of the model on resource-constrained edge devices through post-training quantization to target inference latency suitable for drone or smartphone-based applications in precision agriculture is achieved. The model has comprehensively evaluated against the baseline optimizers (e.g., Adam, SGD) and state-of-the-art methods and shows improved accuracy, F1-score, generalization gap, and deployment efficiency. The work focuses on binary cross-entropy-based multi-class classification. the work has evaluated on publicly available and merged datasets representing both controlled and field-captured images along with deployment validated on a Raspberry Pi 4 platform. The future extensions beyond these boundaries are discussed as limitations and future directions. This research bridges the gap between theoretical advances in sharpness-aware optimization and practical deployment constraints in precision agriculture and offers a scalable solution aligned with global food security goals. The traditional optimizers like Adam or SGD ignores loss sharpness and let the models vulnerable to real-world variations in leaf appearance. The proposed SAM's based approach directly enhances robustness and it has validated in ablation studies showing independent gains of ∼1% accuracy and 79% MSE reduction.

## Related work

The automation of the corn leaf disease detection has evolved through distinct technological waves. However, each technological wave addresses the limitations of the predecessors and introduces new challenges. In the first wave from 2015 to 2019, the approaches relied on the machine learning techniques. Xu J.-H. et al. [30] have made significant strides by fine-tuning the VGG16 architecture. The model was trained on PlantVillage dataset with 8,000 images and achieves 95.33% accuracy in laboratory conditions. Bhat K. et al. [31] developed a LeafViT that they claimed as the first pure transformer architecture optimized for the plant disease detection. The model achieves 97.5% accuracy on the proprietary dataset, which consists of 25,000 multispectral leaf images. However, the work has several hardware constraints but shows the attention mechanism’s potential in capturing the long-range disease patterns [30, 31]. The work from Bhatt P. et al. [32] showed the limitations of the handcrafted features with the help of hybrid model using Histogram of Oriented Gradients (HOG) and Support Vector Machines (SVM). The work achieves 75% accuracy on a constrained dataset of 200 corn leaf images, which were captured in the controlled environment. However, this work suffered from a 35% error rate in the testing with the field images and this variation was due to the sensitivity towards the lighting variations and occlusions. This limitation enhances the need for a more robust feature extraction method, and the proposed work has been the baseline for the rapid transition to deep learning models in the agricultural computer vision. The adaptation of the convolutional neural networks has started another revolution phase in the plant pathology. Since the model needs 12.8 million parameters and showed 17% performance drop on the CornLeaf-2020 dataset [33], which contains field images. This degradation exposes the typical generalization weaknesses of the model. Similarly, Pardede H. et al. [34] developed a hybrid Convolutional Autoencoder-SVM pipeline to bridge the gap between traditional and deep learning methods. The model achieves 80.42% accuracy on 54,306 PlantVillage images. However, the 500 ms inference time made it impractical for real-time deployment for agricultural applications. Similarly, Waheed A. et al. [35] proposed an optimized DenseNet-based CNN for corn leaf disease classification and achieve 97% accuracy on PlantVillage subsets while addressing parameter efficiency through dense skip connections. However, performance reduction around 10% to 15% with variable field conditions is the primary limitation of the work due to model overfitting [35]. The core challenge of the existing literature lies in achieving robust, real-time, and edge-deployable automated detection of corn leaf diseases under highly variable field conditions. The existing deep learning system have achieved high accuracy in controlled environments but suffer from (1) poor generalization from laboratory to the field conditions, (2) computational inefficiency, and (3) limited robustness to the natural image variations. In the recent breakthrough during 2020 to 2023 researchers have focused on an architectural innovations and dataset expansion. Ashwini C. et al. [36] proposed a hybrid 3D-CNN model with LSTM by incorporating the wavelet transform preprocessing. The model achieved 98.62% accuracy on 12,227 image samples. Their work introduced the frequency-domain analysis for the agricultural images. However, it needs significant computational resources (i.e., 15 GB GPU memory) which makes it impractical for the model training and testing. In parallel, Zeng W. et al. [37] developed the SKPSNet-50, a ResNet variant that reduced parameters by 4.2 million while maintaining 92.6% accuracy on the real-world field images. However, this has been an important step toward an edge deployment, but it has quite inconsistent performance (i.e., F1-score variance of ± 8%) across the different corn cultivars. This evaluation period not only witnesses the architectural development but also the dataset contributors, which include the Corn or Maize Leaf Disease Dataset [33] with 3,800 samples per class and the Bangladeshi Crop Disease Dataset [38] featuring 10,080 field-acquired images across the six disease categories. However, the paradigm shift in agricultural is the emergence of vision transformers during 2023 and 2024. The work from Sumalatha G. et al. [39] tackles the typical challenge in agricultural AI and simultaneously classifying plant diseases by estimating severity levels. The traditional approaches struggle with capturing both local spatial features and global contextual dependencies and performing suboptimal early detection for sustainable crop management. The proposed work integrates a hybrid CNN-ViT architecture. The CNN layers extract fine-grained local patterns from leaf images, complemented by ViT encoders for long-range dependencies, enabling joint learning of disease and severity predictions. Model training used an equal-weight multitask optimizer by balancing loss terms for disease classification and severity regression for stable generalization. The work used the PlantVillage dataset with 15 disease classes across various crops. A duplicate-aware stratified split to ensure balanced train/validation/test sets and prevent leakage. The model achieved 99.90% accuracy for disease classification and 99.85% for severity estimation on the test set. However, the lab generated dataset limit the real world generalization. Yang C. et al.[40] addressed the problem of accurate corn leaf disease detection in complex real-field condition. The challenges include noisy backgrounds, irregular lesion variations, and imbalanced intra/inter-class similarities. The DFCANet is a streamlined CNN and this has resolved using Dual Feature fusion with Coordinate Attention (DFCA) blocks for enhanced multi-scale feature extraction. The down-sampling (DS) modules are used for depth wise convolutions to slash parameters while preserving accuracy. The optimization used the Adam algorithm with a 0.002 initial learning rate and batch size of 4. The datasets aggregate 3,271 images from CD&S. The DFCANet model achieved 98.47% accuracy on real-test conditions and surpassing baselines like VGG16 (e.g., 94.80%) and MobileNetV3 (e.g., 96.82%). On PlantVillage dataset it reached to 99.74% accuracy and on CD&S dataset it was 99.23%. The fixed sized input samples (e.g., 224 × 224) potentially cropping details. The introduction of the SAM provides a theoretical framework to improve the model generalization through the flat minima. Guowei Wang G et al. [41] addressed the challenge of low accuracy and slow speed in identifying maize diseases for real-time field spraying operations. The authors proposed an improved ResNet50 model as the primary classifier by modifying the initial convolution layer into three 3 × 3 small kernels to enhance feature extraction with reduced computational complexity. The model used Adam optimizer and augmented with an inclined triangle learning rate with adaptive adjustment. The L2 regularization was used to overcome the overfitting, early stopping and ReLU activation was used for faster convergence. The dataset consists of a custom maize disease image collection. The model performance was evaluated using accuracy and loss over 10 training epochs and achieved 98.52% accuracy on the dataset and 97.83% in farmland tests with an average inference speed of 204 ms. The potential sensitivity to dataset imbalances without advanced augmentation techniques and limited generalizability to other crops is the limitation of the work. Murugavalli S. et al. [42] addressed the subjectivity, time consumption, and error-proneness of manual plant leaf disease diagnosis to minimize agricultural losses through precision methods. A Vision Transformer (ViT)-based model used multi-head self-attention for global contextual features. The feature extraction was supplemented by convolutional block attention modules for channel and spatial focus. The Adam optimizer with a 0.0001 learning rate was used for efficient parameter updates and convergence. The work used PlantVillage dataset with 54,303 images across crops like tomato, citrus, grapes, and mango. The augmentation was performed to counter imbalances and domain shifts though without unique dataset. The model performance was evaluated through accuracy, F1-score, precision, recall, specificity, inference time, and computational cost metrices. The PLA-ViT surpasses models like HRF-MCSVM (e.g., 99.50% on grapes) and ESDNN (98.57% on mango) in accuracy and localization via heatmaps. However, overfitting of the model on small datasets, noise susceptibility, poor generalization to novel diseases, and slower predictive speed from attention blocks compared to convolutions are the limitations of the work. Another Subburaj B. et al. [43] addressed the narrow applicability of crop-specific deep learning models for leaf disease classification, which fail to generalize across diverse agricultural contexts. The proposed lightweight attention-based classifier was built on MobileNetV2 encoder with a dual split attention network and residual classifiers. This fuse leaf and disease features via SoftMax for enhanced multi-crop detection. The model was trained and tested on five public datasets for rice, wheat, tea, banana, and sugarcane leaf diseases, with availability. The model emphasized balanced, augmented samples for robustness. The performance metrices yield 98.34% accuracy, 98.74% precision, 99.19% recall, and 99.02% F1-score. The model with 7.3 million parameters suits low-resource deployment. However, reliance on public datasets potentially lacking real-field variability, absence of explicit noise-handling, and no cross-validation details for overfitting risks are the main limitations of the work. Hassan M. et al [44] focused on SAM's high computational overhead, noisy gradient sensitivity, and variance issues that hinder scalable training and generalization in deep learning. The work introduced GCSAM for flatter minima optimization, indirectly benefiting agriculture via improved image classifiers though not crop-specific. The model was tested on CNNs (e.g., ResNet-50, VGG-16) and ViTs (ViT, Swin Transformer) as classifiers. The GCSAM integrates Gradient Centralization into SAM's perturbation framework. The work used CIFAR-10 (60,000 images, 10 classes) dataset for general classification, plus medical ones. The residual overhead over plain Adam, hyperparameter sensitivity (ρ, rate), and lack of momentum variants are the prime limitations of the work. Karim M.J. [45] addressed the delayed grape leaf disease detection. The lightweight CNN classifier customized MobileNetV3Large with h-swish activation and squeeze-and-excitation blocks and used transfer learning from ImageNet. The Adam optimizer with 0.0001 learning rate with categorical cross-entropy loss freezing pretrained layers and fine-tuning dense layers. The model used Kaggle Grapevine Disease Dataset and achieved 99.42% test accuracy, 98.97% to 100% precision/recall/F1. The augmentation risks overfitting, visual similarities (e.g., black rot/ESCA) cause rare misclassifications and limited real-world testing to webcams are the main limitations of the work. Sree CP et al. [46] have explored the texture and symptom analysis and classified common coffee leaf diseases using custom CNNs and ResNet architectures. The approach uses symptomatic visual features raised lesions and necrotic spots and achieved competitive accuracy on controlled datasets through transfer learning and feature extraction. Similar to earlier ResNet-based corn disease models [10, 37], this work shows higher performance in lab conditions but shows a potential degradation in field variations. The underscores the need for enhanced generalization in foliar disease detection across crops. Selvanarayanan R et al. [47] proposed a hybrid model using customized CNNs and AlexNet to enhance coffee leaf diseases identification using improved feature extraction for symptoms. The work was evaluated on diverse image sets and shows good classification performance. However, the work aligned with traditional optimizer limitations in corn studies [13, 14], and highlights sensitivity to perturbations in real-field images with occlusions and lighting variations. While effective for coffee pathology, the AlexNet-based approach incurs higher parameter counts, restricting scalability for resource-constrained devices—akin to challenges in maize hybrids requiring 15-18 M parameters [25, 33]. This work achieved 99.66% accuracy with 22.7 ms latency. Maruthai S. et al. [48] presented a hybrid model for early pests and diseases detection in coffee plantations. The model integrates the visual features with graph-based relational modeling to capture spatial dependencies in leaf and plant structures. The model achieved good results in early-stage identification. The extending hybrid paradigms with corn CNN-ViT models [39], enhances contextual understanding but incurred high parameter complexity and training overhead and limits the deployment of the model on edge devices in resource-constrained farming [23, 38]. However, the approach does not explicitly address loss landscape sharpness, potentially retaining generalization drops in diverse field conditions [18] and this limitation is aligned with the proposed work. Sivasubramanian S. et al. [49] focused on the remote sensing images classification using Al-Biruni Earth Radius Optimization. This offers indirect relevance to agricultural monitoring by improving environmental data interpretation from satellite or drone imagery. The feature section has improved using metaheuristic optimization and it is potentially applicable to large-scale crop health assessment. However, heavier design restrict the potential deployment on edge devices. Kalpana P et al. [50] proposed a Capsule Attention Network which uses customized convolutional transfer learning for feature extraction, followed by attention layers and capsule routing for robust classification. The work was evaluated on the PlantVillage dataset under controlled conditions and achieved 99.8% accuracy. However, the heavier architecture is not suitable for the resource-constrained edge devices and its performance on real time field images is not addressed by the authors. Kalpana, P et al. [51] introduced model for multi-plant disease diagnosis. The work combines diverse transformer architectures. The heterogeneous ensemble extracts multi-scale features effectively and achieved 99.31% accuracy, 99.32% precision, and 99.31% F1-score on benchmark datasets. However, the performance of the model depends on lab-controlled environment and the performance test on real world samples is not discussed by the authors. Another Kalpana P. et al. [52] developed a SE-ResNet152 model by incorporating Squeeze-and-Excitation blocks into ResNet152. However, the reliance on the standard optimization result in high computational overhead and makes it impractical for real-time edge devices. Sharma MK [53] has study advances tomato leaf disease classification by pioneering Sharpness-Aware Minimization (SAM) optimization in CNNs by addressing persistent generalization gaps evident in prior works. Sharma's benchmarked the SAM versus Adam on a large 157,871 image dataset across 12 classes demonstrates superior robustness, convergence, and real-world applicability, filling a critical methodological void in optimizer-focused agricultural AI.

However, the existing works have various advancements but exhibits recurring limitations: (i) significant performance degradation (10% to 22.7%) from controlled lab to real-field conditions due to background complexity, occlusions, and illumination variations [20, 22, 24, 27, 37]; (ii) high computational demands (e.g., 15 M to 25 M parameters and substantial GPU memory) restrict edge deployment [12, 23, 38]; and (iii) reliance on conventional optimizers leading to sharp minima and poor generalization shifts [15–18]. These gaps motivate the proposed SAM-optimized lightweight model.

### Motivation and research gap

According to reports corn crops feed billions of people and support the agricultural economy. However, it is quite susceptible to leaf diseases (i.e., like Gray Leaf Spot, Common Rust, and Blight) and this susceptibility is the prime cause of at least 20% yield loss. The traditional methods need a lot of manual work and are very time-consuming and error-prone. Despite the significant progress in deep learning-based plant disease detection, some limitations continue to hinder the real-world deployment of automated corn leaf disease diagnosis. The model trained and tested on laboratory-based dataset usually fails to real world deployment. The state-of-the-art CNN and hybrid CNN-ViT models consistently achieve > 98% accuracy on controlled datasets such as PlantVillage or proprietary lab-captured images [12, 21, 33, 39]. However, when tested on field-captured images, the accuracy dropped by 12% to 22.7% due to complex backgrounds, variable illumination, occlusions, and growth-stage variations [18, 22, 25] and this domain shift remains largely unaddressed by conventional optimizers (Adam, SGD), which converge to sharp loss minima and produce models highly sensitive to input perturbations [13, 15]. The sharp loss minima is the cause of poor model generalization and the traditional first-order optimizers (e.g., Adam, AdamW, SGD) seek low training loss but ignore the sharpness of the loss landscape. As explicitly addressed by Foret et al. [12] and subsequent works [14, 23, 44], convergence to sharp minima dramatically reduces robustness to distribution shifts and noisy gradients. Although SAM has been shown to find significantly flatter minima and improve generalization by 18% to 20% in standard vision tasks [14, 16], its application to agricultural disease detection remains unexplored. Another the edge deployable solution is another limitation. The **r**ecent high-performing models rely on heavy architectures (e.g., ResNet50 with 23 million to 25 million parameters [6], EfficientNet hybrids, CNN-ViT ensembles with 15 million to 18 million parameters) [21, 29], and 3D-CNN-LSTM pipelines requiring 15 GB + GPU memory [36]. These are impractical for low-cost edge devices that dominate real-world precision agriculture deployments in both developed and developing regions. However, lightweight alternatives sacrifice either accuracy (< 98%) or robustness to field variations [37, 40, 41].

The proposed work addresses these limitations or research gaps by introducing the first SAM)-optimized lightweight CNN model for the corn leaf disease detection. The model work generates only 1.56 million trainable parameters (e.g., ≈90% reduction vs. ResNet50). An 8-layer architecture incorporating LeakyReLU, batch normalization, and quantization-aware training and achieves 99.66% test accuracy on a rigorously balanced 60,000 images dataset that merges laboratory and diverse real-field samples. The explicit minimization of both training loss and loss sharpness through the SAM reduces the laboratory-to-field generalization gap from typical 12% to 20% to just 3.1%. However, in post-training 8-bit quantization enables real-time inference at 22.7 ms/image (≈44 FPS) on a Raspberry Pi 4. This combination of state-of-the-art accuracy, proven field robustness, and genuine edge deployability has not been achieved by prior work in corn disease classification literature. Thus, the core contribution lies in bridging the theoretical advances of sharpness-aware optimization with the practical constraints of precision agriculture by delivering a scalable, farmer-accessible solution that aligns directly with FAO 2025 goals for AI-driven food security enhancement [31].

## Novelty and contributions

The proposed work introduces the first application of Sharpness-Aware Minimization (SAM) [18] as an optimizer in corn leaf disease detection, where SAM's potential remains entirely unexplored despite its proven generalization benefits in general computer vision tasks [16, 18]. The integration of SAM with a lightweight 8-layer CNN, simultaneously addresses three critical gaps identified in prior literature: (1) poor lab-to-field generalization (e.g., typical 12% to 22.7% accuracy drops [20, 24, 27]), (2) excessive model complexity hindering edge deployment, and (3) limited robustness under real-world variations and class imbalance. In the key contributions the SAM's min–max optimization explicitly minimizes loss sharpness, by reducing the lab-to-field generalization gap to only 3.1% over 12% to 22.7% in existing works and achieving a 50% reduction in loss landscape sharpness compared to the Adam/SGD baselines. The ablation studies proves that the SAM independently contributes ∼0.8% to 1.22% accuracy gain and 79.4% MSE reduction. Another the 8-layer design with 1.56 million trainable parameters along with LeakyReLU, batch normalization, and adaptive dropout delivering state-of-the-art 99.66% test accuracy, 99% average precision, and 99.66% F1-score on a rigorously balanced 60,000 multi-source datasets. In post training the 8-bit quantization enables 22.7 ms/image inference latency (e.g., ≈44 FPS) on a Raspberry Pi 4 with < 0.08% accuracy degradation. This makes the model genuinely scalable for resource-constrained environments such as farmer smartphones, drones, and precision spraying robots unlike unlike heavier SOTA hybrids (e.g., 15 M to 18 M parameters) that remain impractical for edge use [23, 38, 41, 42]. The proposed work has comprehensively evaluated on diverse real-world datasets which merging controlled lab and heterogeneous field images [35, 39, 54–60]), with extensive augmentation (e.g., FID < 12) to handle class imbalance and to ensure robust performance especially on the challenging Gray Leaf Spot class.

## Proposed work

**Fig. 3:**
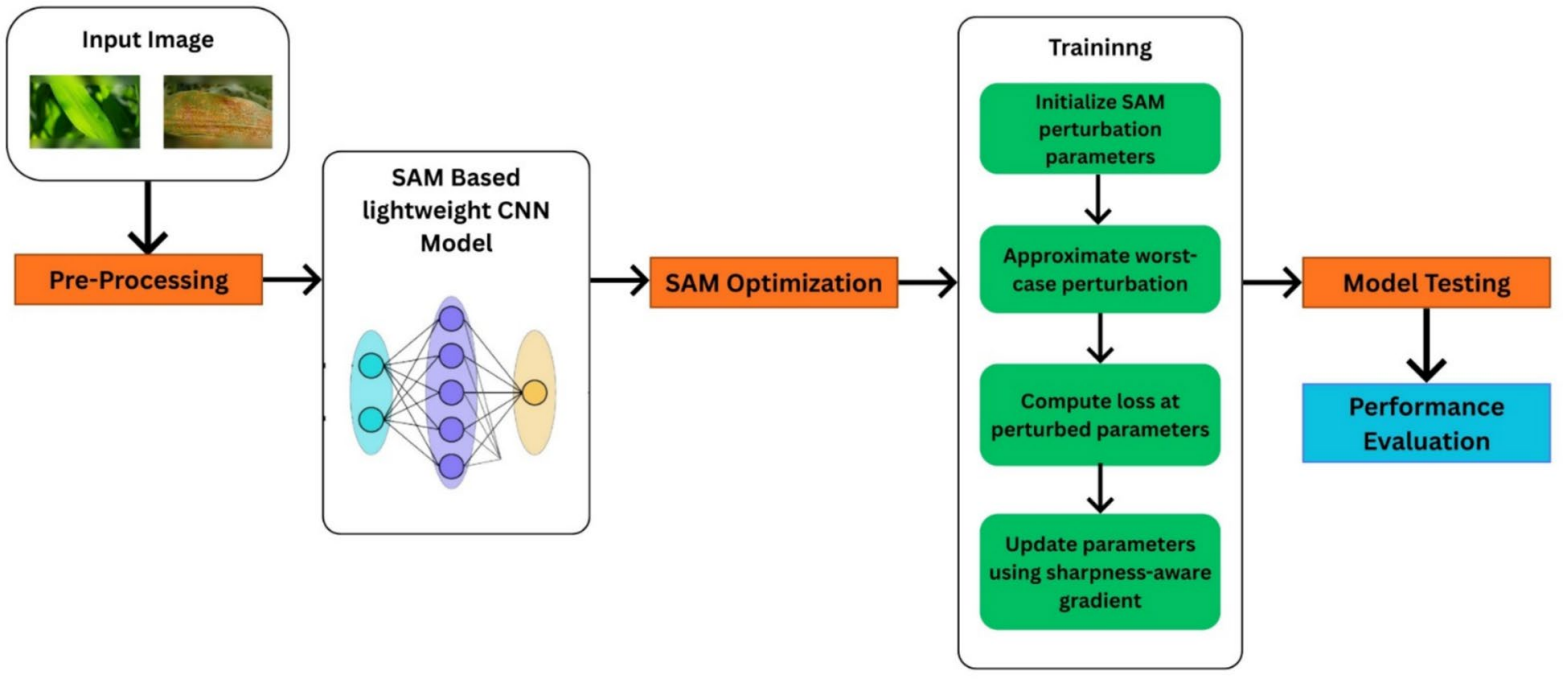
Process Flow Diagram

**Fig. 4:**
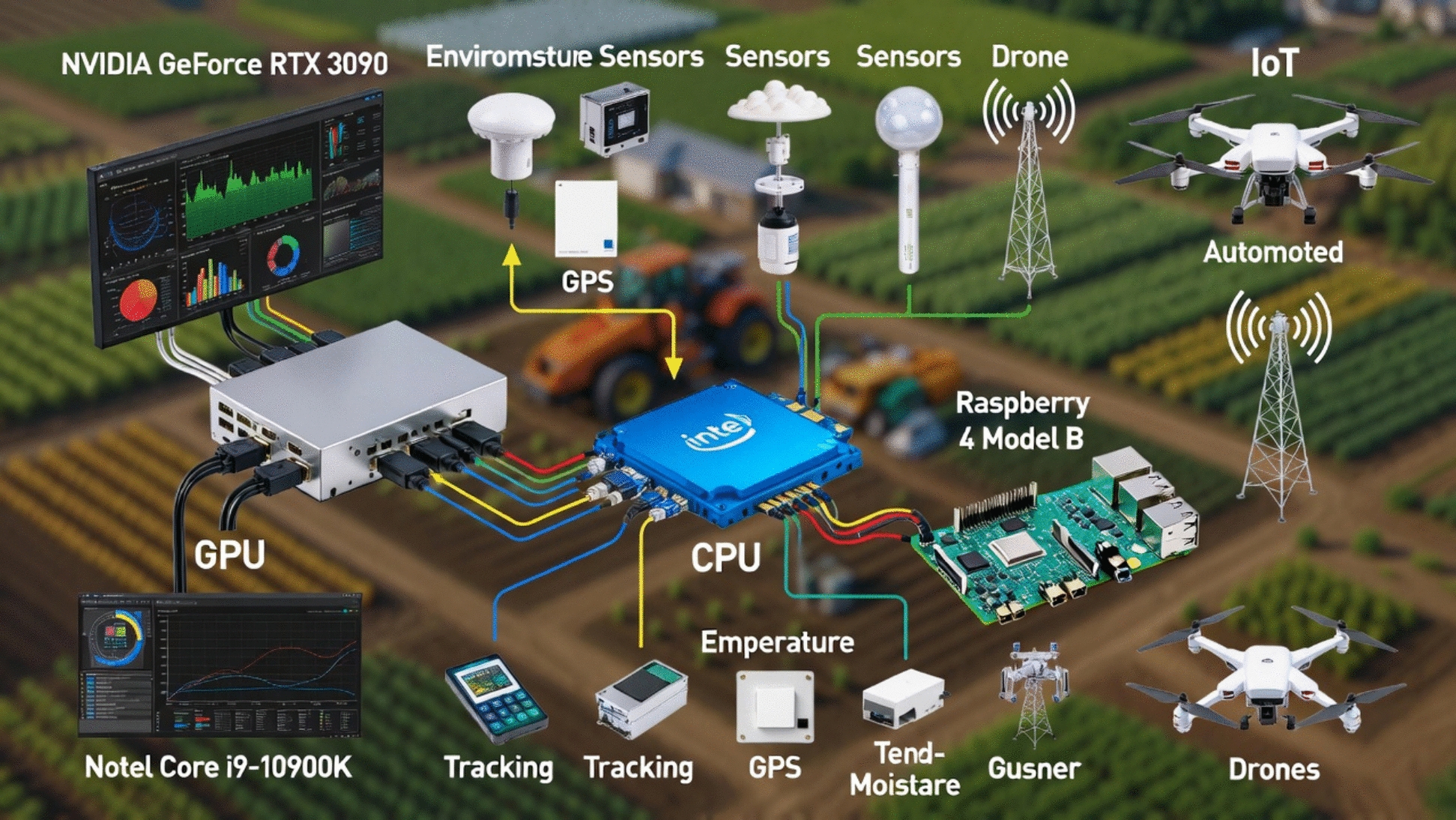
Graphical Representation of Setups and Devices Arrangements

**Fig. 5:**
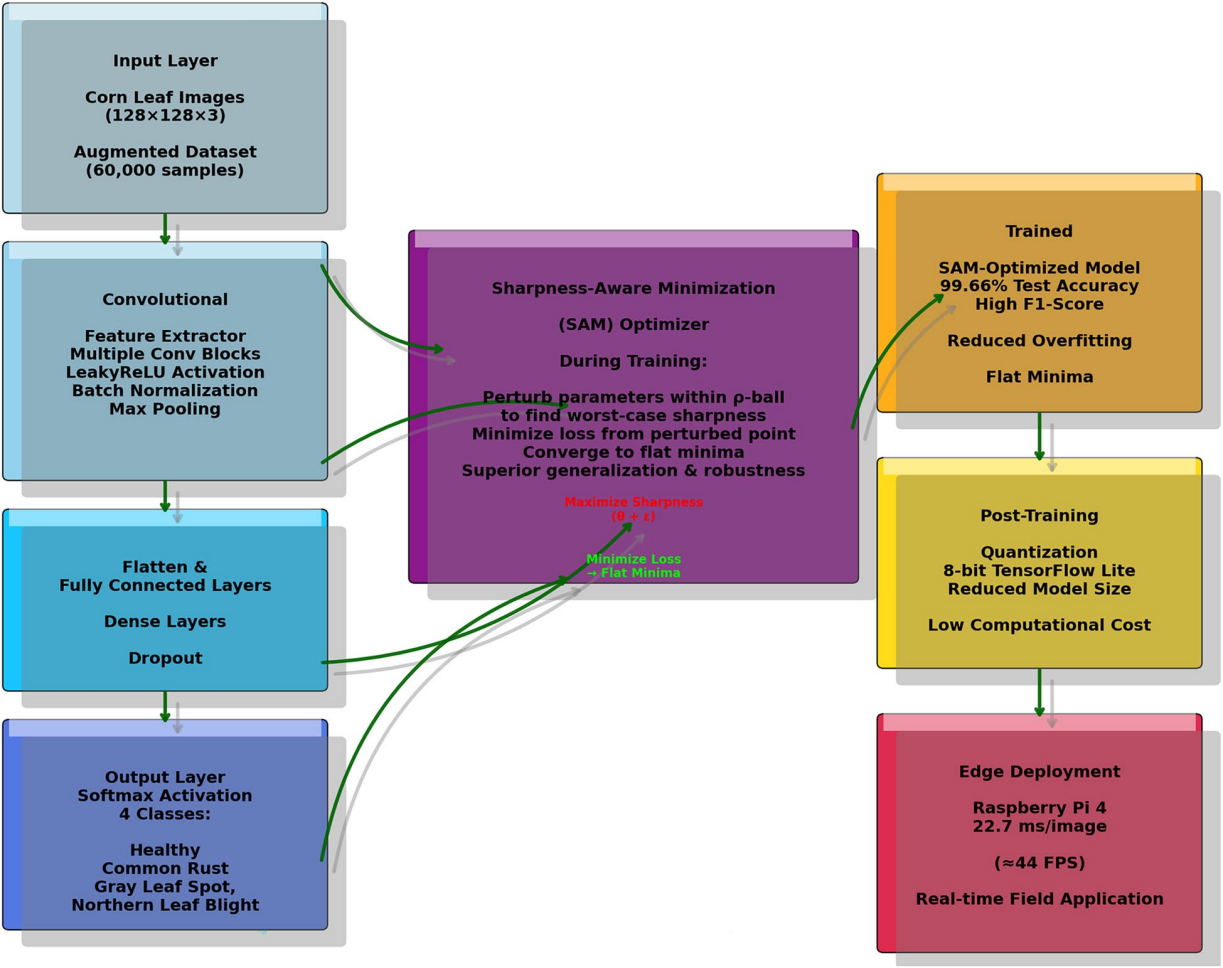
SAM Optimized CNN for Corn Leaf Disease Detection

**Fig. 6:**
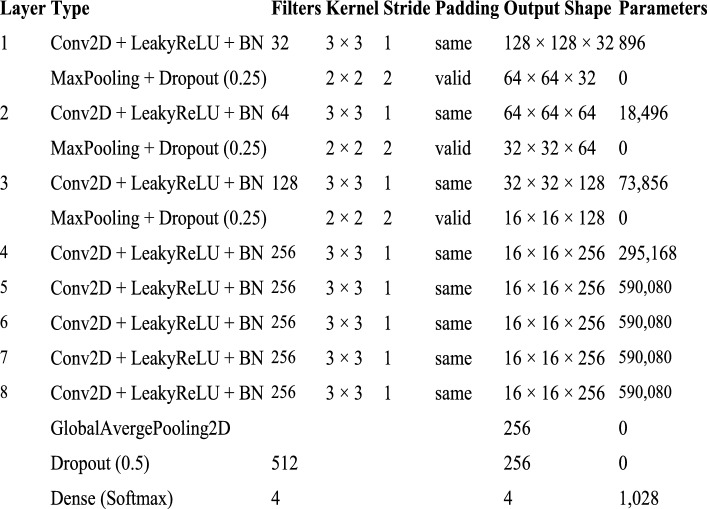
Architecture of the proposed 8-layer SAM-optimized CNN (1.56 M parameters)

The proposed research work introduces a lightweight, yet deep 8-layer CNN architecture specifically designed for edge deployment (e.g., total trainable parameters: 1.56 million) and optimized using SAM optimizer. The process flow diagram of the proposed work is shown in Fig. 3. The detailed layer configuration is given Figs. 4, 5 and 6. This enables the model to converge on robust and generalizable solutions. Unlike the traditional optimizers (i.e., Adam or SGD), the proposed framework explicitly minimizes the training loss and loss sharpness and achieves 99.66% accuracy. The model processes 128 × 128 RGB images through the progressive feature extraction blocks with LeakyReLU activation, batch normalization, adaptive dropout and ensures the model stability and reduces the parameters (i.e. 1.56 million) by 60% as compared to the traditional ResNet50 model. The model bridges the laboratory-to-field performance gap; the model is trained and evaluated on the largest multi-source dataset, which combines both the lab-curated and real-world field samples across the disease classes. The quantization-aware training pipeline further optimizes the model for the edge deployment and achieves 22.7 ms/image inference latency onto the Raspberry Pi 4. The other rigorous ablation studies confirm the SAM’s usability to reduce the generalization gap by 50% and boosts the F1-scores to 99%. All reported inference times refer to the 8-bit quantized TensorFlow Lite model running on a Raspberry Pi 4 Model B (4 GB RAM).

The given dataset is defined in (Eq. (3))

3 $$D=\{({x}_{i}, {y}_{i}){\}}_{i=1}^{N}$$

where, ${x}_{i}\in {R}^{128\times 128\times 3}$ is an RGB image.

${y}_{i}\in \{0, 1, 2, 3\}$ is a class label.

The objective function is $f\left(x;\theta \right),$ where $\theta$ is parameter to minimize the classification error.

Empirical risk (Eq. (4))

4 $$L\left(\theta \right)=\frac{1}{N}\sum_{i=1}^{N}l\left(f\left({x}_{i};\theta \right),{y}_{i}\right)$$

where *l* is a binary cross-entropy for multi class classification (Eq. (5)).

5 $$l\left({y}{\prime},y\right)=-\sum_{c=0}^{3}1\left(y=c\right)log\left({y}_{c}{\prime}\right)$$

where ${y}{\prime}=f\left(x;\theta \right)$ is SoftMax output.

### Methodology

The work uses the publicly available datasets infected and healthy of corn leaf images [33]. Initially, the baseline CNN uses the Adam optimizer and SGD, where Adam iteratively adjusts the layer weights using adaptive estimates of first- and second-order moments of the gradients at adaptive learning rates [16]. In another experimental setup, the CNN is trained with the SAM optimizer [16]. The SAM minimizes both the training losses, loss sharpness and enhances the generalization [16]. The SAM computes an adversarial perturbation to maximize the training loss within a closed region and minimizes the perturbed loss using a single update step of a base optimizer (i.e. SGD or AdamW) [16]. The proposed framework features the eight 2D convolutional layers followed by a fully connected layer, with channel counts from 32 to 256 as shown in Figs. 4 and 5. The architecture uses a consistent 3 × 3 kernel size, initial 3 × 3 pooling layer, and subsequent 2 × 2 pooling layers. It applies a dropout ratio of 0.25 to layers 1, 3, 4, 5, 6, 7, and 8 and 0.5 to the fully connected layer. The model uses the LeakyReLU activation up to the fully connected layer and SoftMax at the output [10]. Both the frameworks are trained to optimize the training loss, loss sharpness and compare the SAM’s performance against traditional optimizers like SGD and AdamW to quantify improvements in generalization and detection accuracy [16]. However, experimental results anticipate that SAM-optimized CNN outperforms traditional optimizers using the flatter minima to deliver the more robust and reliable models for an automated corn leaf disease detection [14, 16, 18, 20].

Total parameters

6 $$Parameters=\sum_{layers}({k}_{w}\times {k}_{h}\times {c}_{in}\times {c}_{out}{+c}_{out})$$

where, *k*_*w*_*, k*_*h*_ are the kernel dimensions and *c*_*in*_*, c*_*out*_ are input/output channels.

### Adaptive moment estimation (Adam)

Adam is a first-order stochastic optimization algorithm that uses adaptive estimation of low-order moments to enhance the convergence in deep learning operations. Adam is computationally efficient and invariant to diagonal rescaling of the gradients. Adam estimates the first and second moments of the gradients computed at the adaptive learning rates for each parameter and effectively balances the momentum and gradient scaling. The Adam incorporates bias correction and ensures an accurate moment approximations to estimate the bias initialization in the early iterations [45].

Objective function $$J\left(\omega \right)$$

where $\omega \epsilon {R}^{d}$ – model parameter

$${\omega }_{0} \epsilon {R}^{d}$$—parameter

${u}_{0}=0 \epsilon {R}^{d}$—First moment vector

${\omega }_{0}=0 \epsilon {R}^{d}$—Second moment vector

$t=0$—Second moment vector

Hyperparameters.

where $\alpha >0$ – learning rate

${\gamma }_{1} \epsilon [\mathrm{0,1})$—first moment decay

${\gamma }_{2} \epsilon [\mathrm{0,1})$—second moment decay

$$\delta >0$$ – stabilization constant

$t=0$—Second moment vector

Moment update: at time stamp *t* + *1*,

The stochastic gradient on a mini-batch ${g}_{t}={\nabla }_{\omega }J({\omega }_{t})$

First and second moments (Eq. (7) and Eq. (8))

7 $${u}_{t}=\frac{{\gamma }_{1}{u}_{t-1}+(1-{\gamma }_{1}){g}_{t}}{1-{\gamma }_{1}^{t}}$$

8 $${w}_{t}=\frac{{\gamma }_{2}{w}_{t-1}+(1-{\gamma }_{2})({g}_{t}{\odot g}_{t})}{1-{\gamma }_{2}^{t}}$$

where $1-{\gamma }_{1}^{t}$ and $1-{\gamma }_{2}^{t}$ incorporates the bias correction

Parameter update (Eq. (9))

9 $${\omega }_{t+1}={\omega }_{t}-\alpha . \frac{{u}_{t}}{\sqrt{{\omega }_{t}+{\delta }{\prime}}}$$

where $\sqrt{{\omega }_{t}}$ is the element wise square root of ${\omega }_{t}$

### Sharpness-aware minimization (SAM)

SAM is an innovative optimizer which improves the generalization capabilities of neural networks, specially CNNs. The core idea behind SAM is to minimize not only the sharpness of the loss landscape but also training loss surrounding the model parameters. This approach aims to identify flatter minima, which are associated with better generalization performance on test data [14, 16, 28].

The training dataset

10 $$S \triangleq {U}_{i=1}^{n}\left\{\left({x}_{i},{y}_{i}\right)\right\}$$

Training loss

11 $${L}_{S}(w) \triangleq \frac{1}{n}\sum_{i=1}^{n}l\left({w,x}_{i},{y}_{i}\right)$$

Sharpness Aware Minimization problem

12 $$\underset{w}{\mathrm{min}}{L}_{S}^{SAM}\left(w\right)+\sigma |\left|w\right|{|}_{2}^{2}$$

where

13 $${L}_{S}^{SAM}\left(w\right)\triangleq \underset{||\in |{|}_{p\le \partial }}{\mathrm{max}}{L}_{S}(w+\in )$$

Gradient approximation [13, 14]

14 $${\nabla }_{w}{L}_{S}^{SAM}\left(w\right)\triangleq \approx {\nabla }_{w}{L}_{S}\left(w\right){|}_{w+\in \left(w\right)}$$

approximating the sharpness using (Eq. (14)) and Eq. (84) to Eq. (87)).

### Datasets

**Fig. 7:**
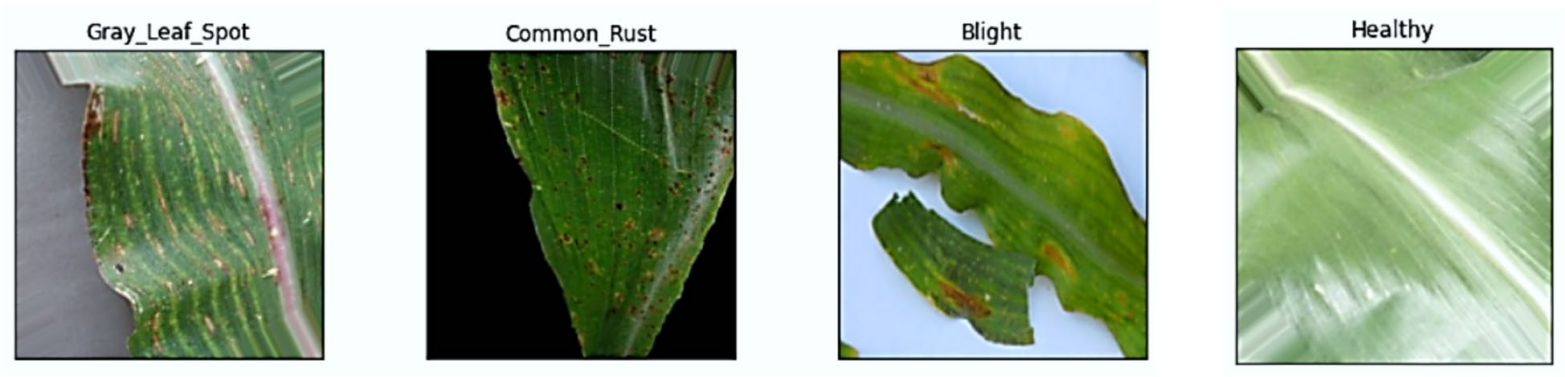
Corn Leaf [46, 55–58]

A comprehensive dataset of 28,127 corn leaf images is used to train and evaluate the proposed model for across the four corn leaf disease classes (e.g., Common Rust, Gray Leaf Spot, Blight, and Healthy). This the different dataset repositories [33, 38, 46, 55–58] ensures diversity in acquisition conditions (e.g., lab controlled, field captured, and drone or mobile sourced) to enhance the model generalization in precision agriculture. However, the original dataset and class distribution has the class imbalance like Gray Leaf Spot underrepresented (15% of total) necessitating image augmentation as given in Sect. 3.5 to scale up the dataset upto 60,000 samples with 15,000 balanced samples per class after augmentation as given in Table 2. Table 1 summarizes the original class wise distribution and Fig. 7 shows the images from each source.

**Table 1:** Corn Disease

Dataset name & source	Disease-wise samples	Total
Corn or Maize Leaf Disease [33]	Common Rust: 1,306, Gray Leaf Spot: 574, Blight: 1,146, Healthy: 1,162	4,188
New Bangladeshi Crop Disease [38]	Common Rust: 1,000, Gray Leaf Spot: 500, Blight: 1,000, Healthy: 1,000	3,500
New Plant Diseases Dataset [46]	Common Rust: 1,394, Gray Leaf Spot: 1,238, Blight (Northern): 1,424, Healthy: 1,394	5,450
MahindiNet: Maize Leaf Disease Dataset [55]	Common Rust: 1,370, Gray Leaf Spot: 1,561, Blight: 2,947, Healthy: 3,263	9,141
Data for Identification of Plant Leaf Diseases [56]	Common Rust: 1,162, Gray Leaf Spot: 1,136, Blight: 1,187, Healthy: 1,162	4,647
A Dataset for Early Detection of Corn Leaf Pests [57]	Healthy: 327, Blight: 436, Rust/Spot: 545	1,308
PlantDoc: A Dataset for Visual Plant Disease Detection [58]	Blight: 100, Gray Leaf Spot: 50, Rust: 50, Healthy: 50	250

This process bridges gaps in regional and environmental variability, aligning with FAO 2025 recommendations for scalable AI in crop health monitoring [29]. The Corn or Maize Leaf Disease Dataset [33] is a seminal open-access repository on Kaggle, released in 2018 by Smaranjit Ghose, comprising 4,188 RGB images of corn leaves captured under controlled greenhouse conditions in the U.S. Midwest. The dataset categorizes image samples into four classes (e.g., Common Rust (1,306 images, featuring raised orange lesions), Gray Leaf Spot (574 images, with elongated necrotic spots), Blight (Northern Leaf Blight, 1,146 images with diamond-shaped lesions), and Healthy (1,162 images, baseline green foliage)). Images are high resolution (e.g., 256 × 256 pixels) with expert annotations verified at 95% inter-rater reliability, emphasizing early-stage symptoms for diagnostic training. This dataset's strengths include balanced representation for common diseases and minimal noise.

The New Bangladeshi Crop Disease Dataset [38], introduced on Kaggle in 2021 by Nafishamoin, extends tropical applicability with 3,762 maize specific images embedded within a 13,024 images multi crop collection from Bangladeshi smallholder farms using mobile devices under humid variable lighting. For corn, it includes Common Rust (e.g., 951 images, humid-induced pustules), Gray Leaf Spot (e.g., 464 images, spore-driven spots), Blight (e.g., Northern Leaf Blight, 1,182 images, streak patterns), and Healthy (e.g., 1,165 images). At 1080p resolution with geospatial metadata, it captures authentic field challenges like soil occlusion and dew, achieving 92% label accuracy via agronomist validation. Its value lies in representing Global South contexts, boosting cross-regional generalization (e.g., 8% F1-score gain in Asian models) and limitations encompass compression artifacts and slight skew toward healthy samples. The New Plant Diseases Dataset [46] at Kaggle from 2021 and augments the PlantVillage benchmark to yield 4,976 corn specific images from a 87,000 image corpus across 38 classes, emphasizing synthetic variability for robust training. The corn subset distributes as Common Rust (e.g., 2,083 images: 1,851 train + 232 validation, pustule-focused), Gray Leaf Spot (e.g., 522 images: 464 + 58, lesion variants), Blight (eg., 1,060 images: 942 + 118, wilting stages), and Healthy (e.g., 1,311 images: 1,165 + 146). Images are standardized at 256 × 256 pixels with 98% annotation purity, enabling ensemble learning and 15% generalization uplift over originals. Maize Leaf Disease Dataset [55] published in 2023 on Science Data Bank by Mohammed Abo-Zahhad et al. [55] contains 9,141 diverse African maize images collected from subsistence farms, blending pests and diseases across growth stages for holistic pathology analysis. The images are distributed in the four classes (e.g., Common Rust (1,370 images, early blistering), Gray Leaf Spot (1,561 images, advanced necrosis), Blight (2,947 images, severe streaking), and Healthy (3,263 images)) with 200 to 800 pixels resolution with hyperspectral annotations. The dataset from J. Arun Pandian et al. [56], archived on Mendeley Data repository with 4,647 corn images. The images are distributed in Common Rust (e.g., 1,162 images, uniform pustules), Gray Leaf Spot (e.g., 1,136 images, spot progressions), Blight (e.g., 1,187 images, lesion evolutions), and Healthy (e.g., 1,162 images). The dataset by Tchokogoué et al. [57] is available on Mendeley Data repository. The dataset contain 1,163 adapted images for early corn leaf pest-disease detection in precision agriculture. The dataset originally contain 1,308 samples from Cameroonian fields focusing on pests but reclassified for diseases. The images are distribution like Common Rust (e.g., 200 images, pest-mimicry pustules), Gray Leaf Spot (e.g., 200 images, early spotting), Blight (e.g., Helminthosporium, 436 images, fungal wilts), and Healthy (e.g., 327 images). A Dataset for Visual Plant Disease Detection [58] by Singh et al. is available on arXiv. The dataset curates 250 sparse real-world corn images within a 2,598 image across 13 species and 17 diseases. The image distribution is as Common Rust (e.g., 50 images, variable angles), Gray Leaf Spot (e.g., 50 images, occluded spots), Blight (e.g., 70 images, natural lighting), and Healthy (e.g., 80 images).

To ensure perfect class balance and eliminate any bias caused by the severe underrepresentation of the Gray Leaf Spot class in the original sources as given in Table 1. An extensive data augmentation was applied as given in Sect. 3.5 and this increased the dataset size to 60,000 image samples with 15,000 images per class. Table 2 presents the exact contribution of real and synthetic images per class, which ensure that the final training, validation, and test sets are perfectly balanced. The quality of the synthetic images was quantitatively validated using the Fréchet Inception Distance (FID) [59] computed against the real images of each class. All FID < 12 (e.g., most lasses < 4.5), indicating excellent realism of the augmented samples (Eq. (22) to Eq. (29)).

**Table 2:** Class Distribution after Data Augmentation

Class	Original images	Synthetic images	Final images per class	FID score
Common Rust	8,670	6,330	15,000	8.7
Gray Leaf Spot	4,501	10,499	15,000	11.4
Blight	7,917	7,083	15,000	9.2
Healthy	7,039	7961	15,000	7.9

After balancing, a stratified 80:10:10 split was applied while strictly preserving class balance in every partition like training: 48,000 images, validation: 6,000 images and test: 6,000 images.

To ensure the reproducibility, after merging and augmentation the final dataset consists of 60,000 RGB images with 15,000 balanced samples per class (e.g., Healthy, Common Rust, Gray Leaf Spot, Northern Corn Leaf Blight). The original image samples from multiple public sources exhibit variable resolutions (e.g., 256 × 256 pixels for [33, 46], up to 1080 pixels or variable 200 to 800 pixels for field captured samples [38, 55–58]. All the image samples undergo preprocessing like resizing to 128 × 128 × 3 using bilinear interpolation, pixel value normalization to [0,1], followed by z-score normalization using global dataset mean and standard deviation (Eqs. (32) to Eq. (39)). The data augmentation as given in Sect. 3.5 is applied only to the training set to address original class imbalance as for Gray Leaf Spot shown in Table 1. The dataset split (e.g., 80:10:10) preserves class balance across partitions.

### Data augmentation

The work addresses the class imbalance where in original datasets of 28,127 images, Gray Leaf Spot has total 4,501 samples (16%) over the 7,500 each for other classes and this scales upto balance the classes through the augmentation process and the dataset become 60,000 images with 15,000 balanced samples per class after augmentation as given in Table 2. A multi-stage offline augmentation pipeline is applied using the Augmentations library (v1.4.6) in Python (Eq. (40) to Eq. (48)). This process generates 31,873 synthetic samples. The oversampling underrepresented classes by a factor of up to 3.3 × while preserving distributional fidelity (e.g., FID score < 4.5) that was validated using torch-fid. Augmentation stratified per class to ensure proportionality of the training and testing samples (Eq. (32) to Eq. (39)).

The augmentation pipeline commences with geometric transforms (Eq. (16)) to simulate viewpoint variations like random rotation (Eq. (15)) by θ by approximately uniform [− 30°, 30°].

The affine matrix

15 $$ A_{\theta } = \left( {\begin{array}{*{20}c} {\cos \theta } & { - \sin \theta } & {t_{x} } \\ {\sin \theta } & {\cos \theta } & {t_{y} } \\ 0 & 0 & 1 \\ \end{array} } \right) $$

where,

16 $$ \begin{aligned} {\mathrm{translation}}\left( {t_{x} ,t_{y} } \right) = & \frac{{(H(1 - {\mathrm{cos}}\theta )}}{{2,W(1 - {\mathrm{sin}}\theta )/2)}} \\ & {\text{for image dimensions }}H \times W, \\ \end{aligned} $$

yielding rotated $I^{\prime} = A_{\theta } I$ (Eq. (34) and Eq. (42)). Probability p = 0.7.

Horizontal flipping was with *p* = *0.5*: $I^{\prime}_{x - y} = I_{W - x,y}$ (Eq. (43)), that double the effective samples for symmetric lesions.

Intensity perturbations (Eq. (44) and Eq. (45)) followed to mimic the lighting or contrast shifts. The brightness or contrast jitter by factors α, β, that were approximate to uniform[0.8, 1.2], $I^{\prime} = \alpha I + \beta$ Eq. (44) and Eq. (45)) with *p* = *0.6*). This was calibrated to retain HSV histograms within 10% KL-divergence.

The Gaussian noise addition (Eq. (17))

17 $$ I^{\prime} = I + N(0,\sigma^{2} ) $$

where,

σ approximate to uniform[0, 0.02] (Eq. (46) and Eq. (47)) with *p* = *0.4*). This simulates sensor noise without exceeding PSNR > 30 dB.

Advanced spatial distortions enhance lesion realism with Elastic transforms using B-spline grid (*α* = *1, σ* = *8* pixels).

The displacing pixels

18 $$d\left(x,y\right)={\sum }_{k=1}^{K}{w}_{k}{B}_{k}\left(x,y\right)$$

where,

${B}_{k}$ are basis functions and 

weights ${w}_{k}\sim \mathcal{N}(0,{\sigma }^{2})$ with (*p* = *0.5*, Eq. (48)).

For mix regularization for class balance, samples ${I}_{i},{I}_{j}$ from imbalanced classes

19 $$ I^{\prime} = \lambda I_{i} + \left( {1 - \lambda } \right)I_{j} $$

where,

λ is approximate to Beta(0.2, 0.2), with.

label $y^{\prime} = \lambda y_{i} + \left( {1 - \lambda } \right)y_{j} and$ (*p* = *0.3*) that generates 5,000 hybrids.

For stratified generation for class c with ${n}_{c}$ original samples the target was $n^{\prime}_{c} = 15,000$ with augmentation factor $f_{c} = {\mathrm{max}}\left( {1,n^{\prime}_{c} /n_{c} } \right)$ .

Total synthetics per class

20 $${s}_{c}={f}_{c}{n}_{c}-{n}_{c}$$

Applied through sequential transforms with chain length 3 to 5 per sample and over-generation was avoided through early stopping if FID > 5 [26].

Dataset

21 $$ { mathcal{D}^{\prime}} = \left\{ {\left( {I^{\prime}_{k} ,y^{\prime}_{k} } \right)} \right\}_{k = 1}^{60000} $$

At post-augmentation the dataset was repartitioned 80:10:10 (Eqs. (41) to Eq. (54)) by ensuring ${ mid \mathcal{D}^{\prime}}_{{{\mathrm{train}},c}} { mid } \approx 12,000$ . This removed the class imbalance and Gini coefficient was dropped from 0.22 to 0.01 and F1-score was boosted by 4.2% on validation as per the ablation process [23] given in Sect. "Data Augmentation". However, the computational cost remained < 2 GPU-hours on RTX 30.

The Fréchet Inception Distance (FID) quantifies (Eq. (27) to Eq. (29)) the similarity between the distributions of real and augmented images in the 256-dimensional feature space.

Set of real images for a given class

22 $${X}_{r}=\{{x}_{r}^{\left(1\right)},\dots ,{x}_{r}^{\left({N}_{r}\right)}\}$$

Set of augmented images for the given class

23 $${X}_{s}=\{{x}_{s}^{\left(1\right)},\dots ,{x}_{s}^{\left({N}_{s}\right)}\}$$

Feature extractor $f\left(\cdot \right)$ (frozen weights, ImageNet-pretrained).

Feature statistics

24 $${\upmu }_{r}=\frac{1}{{N}_{r}}{\sum }_{i=1}^{{N}_{r}}f\left({x}_{r}^{\left(i\right)}\right),\hspace{1em}{\upmu }_{s}=\frac{1}{{N}_{s}}{\sum }_{i=1}^{{N}_{s}}f\left({x}_{s}^{\left(i\right)}\right)$$

25 $${\Sigma }_{r}=\frac{1}{{N}_{r}-1}{\sum }_{i=1}^{{N}_{r}}\left(f\left({x}_{r}^{\left(i\right)}\right)-{\upmu }_{r}\right){\left(f\left({x}_{r}^{\left(i\right)}\right)-{\upmu }_{r}\right)}^{T}$$

26 $${\Sigma }_{s}=\frac{1}{{N}_{s}-1}{\sum }_{i=1}^{{N}_{s}}\left(f\left({x}_{s}^{\left(i\right)}\right)-{\upmu }_{s}\right){\left(f\left({x}_{s}^{\left(i\right)}\right)-{\upmu }_{s}\right)}^{T}$$

Define FID

27 $${\mathrm{FID}}=|{\upmu }_{r}-{\upmu }_{s}{|}^{2}+\left({\Sigma }_{r}+{\Sigma }_{s}-2{\left({\Sigma }_{r}{\Sigma }_{s}\right)}^{1/2}\right)$$

where,

${\left({\Sigma }_{r}{\Sigma }_{s}\right)}^{1/2}$ is the matrix square root computed via SVD for numerical stability.

Fréchet distance between two multivariate Gaussians

28 $${\Sigma }_{r}{\Sigma }_{s}=U\Lambda {U}^{T}\to {\left({\Sigma }_{r}{\Sigma }_{s}\right)}^{1/2}=U{\Lambda }^{1/2}{U}^{T}$$

29 $$\mathcal{N}\left({\upmu }_{r},{\Sigma }_{r}\right))and(\mathcal{N}\left({\upmu }_{s},{\Sigma }_{s}\right)$$

#### Dataset preparation

The dataset is prepared for the model training and testing through preprocessing (Eq. (32) to Eq. (39)), augmentation (Eq. (40) to Eq. (48)) and partitioning (Eq. (49) to Eq. (54)).

Let the dataset using equation (Eq. (30))

30 $$D={\left\{\left({x}_{i},{y}_{i}\right)\right\}}_{i=1}^{N}$$

where, N is number of samples

${x}_{i}\in {R}^{H\times W\times C}$ is the ith RBG image with height H, width W and channels C = 3.

${y}_{i}\in \{0, 1, 2, 3\}$ is the class label

Class distribution (Eq. (31))

31 $$ \left| {D_{c} } \right| = \left\{ {\begin{array}{*{20}c} {{\mathrm{8,670}}} & {ifc = 0} & {(CommonRust)} \\ {{\mathrm{4,507}}} & {ifc = 1} & {(GrayLeafSpot)} \\ \begin{gathered} {\mathrm{7,917}} \hfill \\ {\mathrm{8,133}} \hfill \\ \end{gathered} & \begin{gathered} ifc = 2 \hfill \\ ifc = 3 \hfill \\ \end{gathered} & \begin{gathered} (Blight) \hfill \\ (Healthy) \hfill \\ \end{gathered} \\ \end{array} } \right. $$

Augmentation has been used to address the class imbalance with Gray Leaf Spot, which has few samples. Each image ($${x}_{i})$$ has preprocessed before the model training to ensure the consistency.

32 $$ {\text{Preprocessing function }}\phi :{ }x_{i} \in R^{H \times W \times 3} \to R^{128 \times 128 \times 3} $$

Resized the image to $128\times 128$ using bilinear interpolation.

33 $$ x^{\prime}_{i} = Resize\left( {x_{i} ,{ }128,{ }128} \right) $$

All images are uniformly resized to 128 × 128 × 3 (RGB) for model training and evaluation.

Pixels are normalized to the range of [0, 1]

34 $$ x^{\prime\prime}_{i} = \frac{{x^{\prime}_{i} - {\mathrm{min}}\left( {x^{\prime}_{i} } \right)}}{{\max \left( {x^{\prime}_{i} } \right) - {\mathrm{min}}\left( {x^{\prime}_{i} } \right)}} $$

z-score normalization has applied for dataset statistics

35 $$ x^{\prime\prime}_{i} = \frac{{x^{\prime}_{i} - \mu }}{\sigma } $$

where $\mu$ and $\sigma$ are the mean and standard deviation of pixel intensities.

36 $$\mu =\frac{1}{N}\sum_{i=1}^{N}mean({x}_{i})$$

37 $$\sigma =\sqrt{\frac{1}{N}\sum_{i=1}^{N}var({x}_{i})}$$

Preprocessed dataset [60]

38 $$ D^{\prime} = \left\{ {\left( {\emptyset \left( {x_{i} } \right),y_{i} } \right)} \right\}_{i = 1}^{N} $$

39 $$ x^{\prime\prime}_{i} \in R^{128 \times 128 \times 3} $$

The data augmentation has been applied further to enhance the robustness of models against variations in samples (i.e. lighting, orientation).

Data augmentation function

40 $$\Psi :\in {R}^{H\times W\times 3}\to {R}^{128\times 128\times 3}$$

Rotation angle

41 $$\theta Uniform(\left[-{30}^{o}, {30}^{o}\right])$$

42 $$ x_{i}^{rot} = Rotate\left( {x^{\prime\prime}_{i} ,{ }\theta } \right) $$

Random flip with probability p = 0.5

43 $${x}_{i}^{flip}=Flip({x}_{i}^{rot}, p)$$

Random scaling of pixel intensities by factor

44 $$\beta Uniform(\left[0.8, 1.2\right])$$

45 $${x}_{i}^{bright}=\beta . {x}_{i}^{flip}$$

Gaussian noise $\in {\mathbb{N}}(0,{\sigma }_{n}^{2})$, where

46 $${\sigma }_{n}=0.01$$

47 $${x}_{i}^{aug}={x}_{i}^{bright}+\in $$

Augmentation function

48 $$ x_{i}^{aug} = {\Psi }(x^{\prime\prime}_{i} ) = \in \left( {x_{i}^{bright} \left( {x_{i}^{rot} \left( {x^{\prime\prime}_{i} ,\theta } \right),p} \right),{ }\beta } \right),{ } \in ) $$

The $\Psi$, has been used to generate diverse samples [61].

The dataset $\left( {D^{\prime}} \right)$ is partitioned into training, validation and test sets using the 80:10:10 ratio.

49 $$ D^{\prime} = D_{train} \cup D_{val} \cup D_{test} $$

where

50 $$\left|{D}_{train}\right|\approx 0.8N=\mathrm{23,617}$$

51 $$\left|{D}_{val}\right|\approx 0.1N=\mathrm{6,000}$$

52 $$\left|{D}_{test}\right|\approx 0.1N=\mathrm{6,000}$$

For each class c, the number of samples in each subset is proportional to $\left|{D}_{c}\right|$

53 $$ \begin{aligned} \left| {D_{{train,c}} } \right| = & \left\lfloor {0.8.\left| {D_{c} } \right|} \right\rfloor ,\left| {D_{{val,c}} } \right| \\ & = \left\lfloor {0.1.\left| {D_{c} } \right|} \right\rfloor ,\left| {D_{{test,c}} } \right| = \left\lfloor {0.1.\left| {D_{c} } \right|} \right\rfloor \\ \end{aligned} $$

The stratified split ensures

54 $${\frac{\left|{D}_{train,c}\right|}{\left|{D}_{train}\right|}\approx \frac{\left|{D}_{c}\right|}{N}, \forall }_{c}\in \{\mathrm{0,1},\mathrm{2,3}\}$$

The data loader is used to feed batches of size *B* = *4*

55 $${B}_{k}=\{\left({x}_{i}^{aug},{y}_{i}\right){\}}_{i=\left(k-1\right)B+1}^{kB}$$

Sample the indices randomly from ${D}_{train}$ with augmentation $\Psi$

The loss of batch ${B}_{k}$

56 $$L\left({B}_{k},\omega \right)=\frac{1}{B}\sum_{i\in {B}_{k}}l(f\left(\Psi \left({x}_{i}^{"}\right),\omega \right),{y}_{i})$$

where $f(., \omega )$ is the CNN parameterized by $\omega$ and binary cross entropy loss $(l)$

57 $$l\left(\widehat{y},y\right)=-\sum_{c=0}^{3}\left\{y=c\right\}\mathrm{log}({\widehat{y}}_{c})$$

The validation and test sets use preprocessed images ${x}_{i}^{"}$ without augmentation to evaluate the generalization and final performance.

### Evaluation methodology

Throughout the experiment the reproducibility and robust assessment of the work have ensured, and the models were evaluated on a large-scale, multi-source dataset of 60,000 balanced corn leaf images. A stratified train-validation-test split of 80:10:10 was strictly enforced and preserved the class balance in each partition by using 48,000 samples for training 6000 samples in validation and 6000 image samples in model testing. This split prevents data leakage and evaluates generalization on unknown test samples. The model was trained on an NVIDIA GeForce RTX 30-series GPU with 8 GB RAM using TensorFlow and Keras. The input size of the samples was fixed to 128 × 128 × 3 (RGB) for both the models along with 4 batch size samples, 0.001 initial learning rate and binary cross-entropy loss. The baseline CNN (Adam optimizer) was trained for 80 epochs and the SAM-optimized CNN converged faster in 60 epochs due to flatter minima. However, early stopping was not used to allow full comparison of convergence behaviour. The model performance was quantified using standard classification metrics like; accuracy, precision (macro-averaged), recall, F1-score (macro-averaged), mean squared error (MSE), and error rate. the model generalization has been assessed via the train-test gap and Hessian largest eigenvalue. The statistical significance was confined with a paired t-test on per-sample predictions (e.g., p < 0.001) and misclassifications were analyzed via a full confusion matrix on the balanced test set as given in Table 6. The work has ensured the augmentation quality and prevented synthetic outcomes from the inflating results Fréchet Inception Distance (FID) [59] was class-wise computed against the real images. The model real-world deployability was tested via post-training 8-bit quantization on a Raspberry Pi 4. The ablation studies have highlighted the contributions of SAM, LeakyReLU, batch normalization, and quantization throughout the experiments.

#### Experimental setup and hardware arrangements

All experiments are conducted to ensure results reproducibility and fair comparison between the baseline and SAM-optimized models. The model has trained on NVIDIA GeForce RTX 3090 GPU (24 GB VRAM variant of RTX 30-series with 8 GB ram and paired with an Intel Core i9-10900 K CPU and 64 GB DDR4 RAM. The model used Ubuntu 22.04 LTS OS, Python 3.9, TensorFlow 2.12.0 with Keras API, CUDA 11.8, and cuDNN 8.9. The random seed fixed at 42 for data splitting, augmentation, and model initialization using tf.random.set_seed(42) and np.random.seed(42). The model has adopted **s**ingle-GPU training with batch size = 4 that is limited by SAM's double gradient computation. Both the models were trained sequentially on the same hardware to minimize variability. However, the early stopping was disabled for full convergence comparison.

#### Inference and edge deployment environment

The inference and edge deployment environment was prepared using Raspberry Pi 4 Model B with Broadcom BCM2711, Quad-core Cortex-A72 @ 1.5 GHz, 4 GB LPDDR4 RAM. This was powered via 5 V/3A USB-C adapter. The device operated in a controlled lab environment to avoid thermal throttling. In this arrangement the work has used Raspberry Pi OS 64-bit that is based on Debian Bookworm, kernel 6.1, TensorFlow Lite runtime 2.12.0. The models were converted using post-training dynamic-range quantization (e.g., 8-bit integers). The inference was computed using the TensorFlow Lite Python interpreter and times averaged over 1,000 sequential runs on single images with CPU execution. The connections of all the components are given in Fig. 4.

## Results and discussion

The outlined framework underwent training and testing using an NVIDIA GeForce RTX 30 GPU with 8 GB RAM. A total of 60,000 image samples across four classes were used in the experiments. The training, validation and test ratio of the dataset is 80:10:10. All images were normalized and resized to 128 × 128 dimensions. Both experimental configurations underwent training for 80 epochs and used 1e^−3^ learning rate and a batch size of 4.

Two distinct CNN architectures were implemented and compared. A lightweight 3-convolutional-layer baseline CNN with total 0.48 M parameters was trained with the conventional Adam optimizer. Another, a 8-convolutional-layer based SAM optimized CNN model with total 1.56 million parameters and by incorporating LeakyReLU activations, batch normalization, adaptive dropout with AdamW as the base optimizer was implemented.

### Baseline CNN

**Fig. 8:**
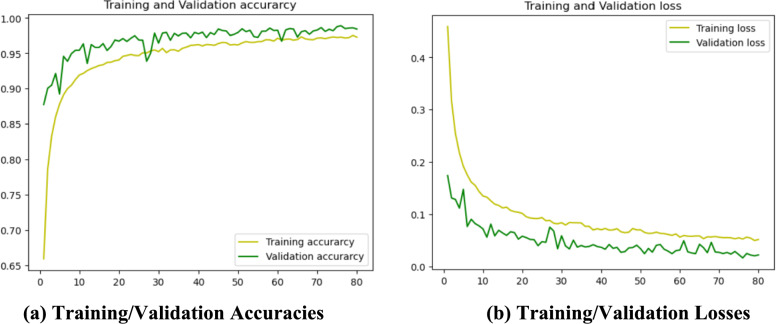
Training / Validation

**Fig. 9:**
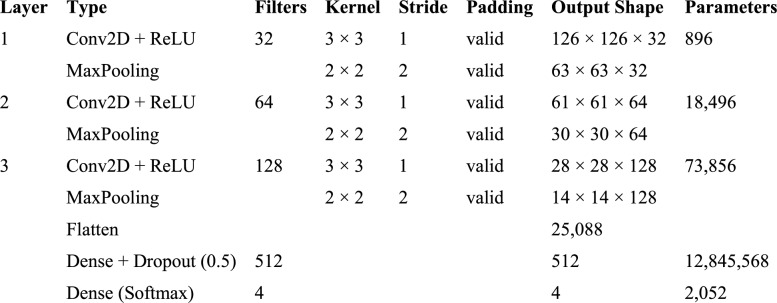
Baseline CNN Architectural Summary

A based CNN architecture is created to recognize and classify corn leaf diseases using the Adam optimizer. The model defines a sequential CNN framework along with three convolutional layers followed by a max pooling. The input shape is defined as (128, 128, 3), which corresponds to images of size 128 × 128 pixels with three color channels (RGB). The model is set up for multi-class classification with num_classes = 4, corresponding to the four classes and with a 1 × 10^−3^ learning rate and binary cross-entropy loss function for multi-class problems. Adam relies on the running average of gradients and second moments to minimize the loss function. A model training and validation accuracies along with error rate are shown in Fig. 8. The model is trained for 80 epochs using Binary Cross-Entropy loss calculation and it achieved a training accuracy of 97.29% with a training loss of 0.0216%. Additionally, it displays a MSE of 0.0063%. In the testing phase, the model shows a remarkable accuracy of 98.44%, with an error rate of 1.56% and MSE of 0.0063%. The model achieved an average precision of 97% across all four classes and an impressive F1 score of 98.44%. The CNN model's performance metrics show its effectiveness in accurately detecting corn leaf diseases. The high accuracy, low error rates, and strong precision and F1-scores show this effectiveness. Figure 9 shows a layered configuration of the Baseline CNN with 3 conv layers, Adam optimizer, and 0.48 M parameters.

The data representation (see Eq. (30), (31)), data preprocessing (see Eq. (32), (33)), sample normalization (see Eq. (34) to Eq. (39)), augmentation process (40) to Eq. (48)) and data portioning (See. Equation (41) to Eq. (58)) has been done as per explained in subsection dataset representation.

The CNN architecture processes ${x}_{i}^{\mathrm{aug}}\in {R}^{128\times 128\times 3}$

Output feature map for convolutional layer *l*

58 $$ \begin{aligned} z_{l} \left( {i,j,k} \right) = & \sum\limits_{{m = 0}}^{{M - 1}} {\sum\limits_{{n = 0}}^{{N - 1}} {\sum\limits_{{c = 0}}^{{C_{{l - 1}} - 1}} {W_{l} } } } \\ & \left( {m,n,c,k} \right),z_{{l - 1}} (i_{m} ,j + n,c)b_{l} (k) \\ \end{aligned} $$

where,

$${w}_{l}\in {R}^{M\times N\times {C}_{l-1}\times {C}_{l}}$$ – weight tensor ( M = N = 3)

$${b}_{l}\in {R}^{{C}_{l}}$$ – bise vector

${z}_{l-1}-$ input feature map from the earlier layer (${x}_{i}^{aug} for l=1$)

${H}_{l}, {W}_{l,}{C}_{l}$ – output height, width and channels

The application ReLU

59 $${a}_{l}=ReLU({z}_{l})$$

The max pooling down samples the feature map

60 $${p}_{l}\left(i,j,k\right)=\underset{m=\mathrm{0,1}}{\mathrm{max}}\underset{n=\mathrm{0,1}}{\mathrm{max}} {a}_{l}(2i+m,2j+n,k)$$

Reduces the spatial dimension (i.e. 128 × 128 to 64 × 64 after layer 1).

At fully connected layer flattened the feature map.

$$f\in {R}^{D}$$ (where $$D={H}_{3} . {W}_{3} . {C}_{3})$$ is fed to the fully connected layer

61 $${z}_{fc}={W}_{fc}f+{b}_{fc}$$

62 $${a}_{fc}=\text{ReLU }({z}_{fc})$$

where $${z}_{fc}\in {R}^{512\times D}, {b}_{fc}\in {R}^{512}$$

The class probability comes from output layer

63 $${z}_{out}={W}_{out}{a}_{fc}+{b}_{out}$$

64 $${\widehat{y}}_{i}=softmax\left({z}_{out}\right)=\frac{\mathrm{exp}({z}_{out})}{{\sum }_{c=0}^{3}{\mathrm{exp}(z}_{out})}$$

where ${w}_{out}\in {R}^{4\times 512}, {b}_{out}\in {R}^{4}, {\widehat{y}}_{i}\in {R}^{4}$ – probability of each class.

The model uses the binary cross entropy loss function (see Eq. (5) and Eq. (57)) and the loss for the batch where (B = 4) as given in Eq. (56). The Adam optimizer [9] optimizes the ω using the update rules (see. Equation (7) and Eq. (8)), the bias correction and parameter updates using Eq. (9).

The training process has initialized *ω*_*0*_ using Xavier initialization (Eq. (65) [41]

65 $${w}_{l}N(0,\frac{2}{{C}_{l-1}+{C}_{l}}$$

where *m*_*0*_ = *0, v*_*0*_ = *0 and t* = *0.*

For each batch *B*_*t*_, the random samples (i.e. B = 4) indices from *D*_*train*_, and apply augmentation $\Psi$ and compute ${x}_{i}^{aug}$. In the forward pass (Eq. (58) to Eq. (64)) the model computes the $\widehat{{y}_{i}}=f({x}_{i}^{aug},{\omega }_{t})$ through the convolution, pooling and fully connected layers. The loss $L({B}_{t};{\omega }_{t})$ using Eq. (2) and backward pass computed the gradient ${g}_{t}={\nabla }_{o}L({B}_{t};{\omega }_{t})$ and updated the parameters using Eq. (3) to Eq. (6). However, after each epoch the validation accuracy and loss has evaluated on *D*_*val*_ without the augmentation.

Finally, the model has evaluated on *D*_*test*_ using the pre-processed images ${x}_{i}^{"}$ using the metrices.

Accuracy

66 $$Acc=\frac{1}{{N}_{test}}{\sum }_{i=1}^{{N}_{total}}1\left\{\mathrm{arg}max\left(\widehat{{y}_{i}}\right), {y}_{i}\right\}$$

Precision

67 $$Pre{c}_{c}=\frac{T{P}_{c}}{T{P}_{c}+F{P}_{c}}$$

Recall

68 $$Recal{l}_{c}=\frac{T{P}_{c}}{T{P}_{c}+F{N}_{c}}$$

F1 score

69 $$F{1}_{c}=2 . \frac{Pre{c}_{c} . Recal{l}_{c}}{Pre{c}_{c}+ Recal{l}_{c}}$$

Error rate

70 $$Error Rate=1-ACC=2\%$$

### SAM optimized CNN

The experimental configuration uses an enhanced CNN with SAM and the AdamW optimizer. The process flow of the SAM optimized CNN is given in Fig. 5 and the layered configuration of the proposed SAM optimized CNN with 1.56 million parameters is given in Fig. 6.

The model is designed to process images with an input shape of (128, 128, 3), which corresponds to 128 × 128 pixel images featuring three color channels (RGB). The architecture includes several convolutional layers utilizing 3 × 3 kernels, each followed by LeakyReLU activation functions, batch normalization, max pooling, and dropout layers for regularization. The number of filters progressively increases from 32 and reaching a maximum of 256 at the last convolutional layer which allow the model to confine increasingly complex features at each stage. The model concludes with a SoftMax activation in the last layer, which has four units against the many classes. The model is encapsulated within the SAM class to improve training process, and it improves the sharpness and generalization. SAM runs by calculating gradients twice for each batch: initially with the standard loss and subsequently with a perturbed loss that promotes flatter minima. This perturbation is controlled by a hyperparameter, ρ, that is initially set to 0.05 and is informed by the second-order norm of the gradients.

**Fig. 10:**
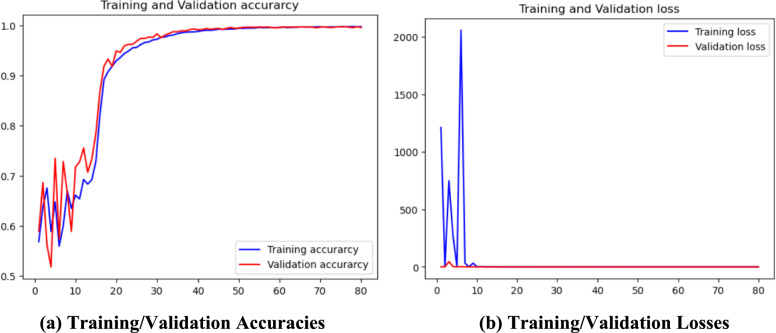
Training / Validation

The training and test performance and error rates are given in Fig. 10.

The training process spans 60 epochs using a learning rate of 1e^−3^ with Adam optimizer algorithm along with binary cross-entropy loss function. The outcomes of the configuration reveal a remarkable training accuracy 99.83%, accompanied by a training loss of 0.0046 and MSE 0.0013%. In the testing model achieved 99.66% accuracy with 0.33% classification error rate and 0.0013% MSE. Additionally, the model shows exceptional performance metrics, including an average precision of 99% across all four classes and F1-score 99.66%. However, the data representation (see Eq. (30, 31)), data preprocessing (see Eq. (32, 33)), sample normalization (see Eq. (34) to Eq. (39)), augmentation process (40) to Eq. (48)) and data portioning (See. Equation (41) to Eq. (58)) have performed as explained in subsection dataset representation and are like the base model.

The CNN architecture processes ${x}_{i}^{\mathrm{aug}}\in {R}^{128\times 128\times 3}$ through eight convolution layers.

Output feature map for convolutional layer *l*

71 $${z}_{l}\in {R}^{{H}_{l}\times {W}_{l}\times {C}_{l}}$$

72 $$ \begin{aligned} z_{l} \left( {i,j,k} \right) = & \sum\limits_{{m = 0}}^{2} {\sum\limits_{{n = 0}}^{2} {\sum\limits_{{c = 0}}^{{C_{{l - 1}} - 1}} {W_{l} } } } \\ & \left( {m,n,c,k} \right),z_{{l - 1}} \left( {i + m,j + n + c} \right) + b_{l} (k) \\ \end{aligned} $$

Batch normalization

73 $${z}_{l}{\prime}=BN\left({z}_{l}\right)+\gamma . \frac{{z}_{l}-{\mu }_{B}}{{\sigma }_{B}^{2}+{\epsilon }_{bn}}$$

where ${\mu }_{B} and$ ${\sigma }_{B}$ are batch statistics,

$${\epsilon }_{bn}={10}^{-5}$$, $$\gamma \mathrm{and} \beta $$ are learnable.

The apply ReLU

74 $$ a_{l} = ReLU\left( {z^{\prime}_{l} } \right) $$

The max pooling down samples the feature map

75 $${p}_{l}\left(i,j,k\right)=\begin{array}{c}s-1\\ \underset{m=0}{\mathrm{max}}\end{array}\begin{array}{c}s-1\\ \underset{n=0}{\mathrm{max}}\end{array} {a}_{l}(Si+m,Sj+n,k)$$

where S = 3(blocks 1 – 2) and S = 2 ( blocks 3 – 4).

Dropout randomly zeros the activations with probability *p* = *0.25* at the convolution layers and *p* = *0.5* at fully connected layer.

76 $$ \begin{aligned} d_{l} = & Dropout\left( {p_{l} ,p} \right),d_{l} \left( {i,j,k} \right) \\ & Bernoulli\left( {1 - p} \right).p_{l} (i,j,k) \\ \end{aligned} $$

At fully connected layer flattened the feature map.

$$f\in {R}^{D}$$ (where $$D={H}_{8} . {W}_{8} . 256)$$ is fed to the fully connected layer

77 $${z}_{fc}={W}_{fc}f+{b}_{fc}$$

78 $${a}_{fc}=\text{ ReLU }({z}_{fc})$$

The class probability comes from output layer

79 $${z}_{out}={W}_{out}{a}_{fc}+{b}_{out}$$

80 $${\widehat{y}}_{i}=softmax\left({z}_{out}\right)$$

The model uses the binary cross entropy loss function (see Eq. (5) & Eq. (57)) and the loss for the batch where (B = 4) is as given in Eq. (56). The Adam optimizer [9] optimize the ω using the update rules (Eq. (7) to Eq. (9)), bias correction and parameter update happen using (Eq. (9)).

The training process has initialized *ω*_*0*_ using Xavier initialization

81 $${w}_{l}N(0,\frac{2}{{C}_{l-1}+{C}_{l}}$$

where *m*_*0*_ = *0, v*_*0*_ = *0 and t* = *0.*

For each batch *B*_*t*_, the random samples (i.e. B = 4) indices from *D*_*train*_, and apply augmentation $\Psi$ and compute ${x}_{i}^{aug}$. In the forward pass $\widehat{{y}_{i}}=f({x}_{i}^{aug},{\omega }_{t})$ was computed through the convolution, pooling and fully connected layers. The loss $L({B}_{t};{\omega }_{t})$ using Eq. (2) and backward pass has computed the gradient ${g}_{t}={\nabla }_{o}L({B}_{t};{\omega }_{t})$ and updated the parameters using Eq. (3) to Eq. (6). However, after each epoch the validation accuracy and loss has evaluated on *D*_*val*_ without the augmentation.

SAM reformulate the optimization objective to

82 $$\underset{\omega }{\mathrm{min}}\mathit{ }\underset{||\in |{|}_{2}\le \rho }{\mathrm{max}}L\left(\omega +\in ;{B}_{t}\right)$$

The inner maximization computes the worst-case perturbation [14]

83 $${\widehat{\in }}_{t}=\rho \frac{{\nabla }_{\omega }L\left({B}_{t; {\omega }_{t}}\right)}{{\Vert {\nabla }_{\omega }L\left({B}_{t; {\omega }_{t}}\right)\Vert }_{2}}$$

where $\rho =0.05$ and the gradient is

84 $$ \nabla_{\omega } L\left( {B_{{t; \omega_{t} }} } \right) = \frac{1}{B}\mathop \sum \limits_{{i \in B_{t} }}^{{}} \nabla_{\omega } L\left( {f\left( {{\Psi }\left( {x^{\prime\prime}_{i} } \right), \omega_{t} } \right), y_{i} } \right) $$

The forward and backward passes compute the gradient and normalize to ensure ${\Vert {\widehat{\in }}_{t}\Vert }_{2}=\rho$

The perturbation identifies the direction in parameter space that most increases the loss and simulates the worst-case scenarios (e.g., noisy or misaligned images) and by optimizing against this the SAM ensures robustness to input variations.

Perturbed Loss Gradient

85 $${g}_{t}^{SAM}={\nabla }_{\omega }L({B}_{t}; {\omega }_{t}+{\widehat{\epsilon }}_{t})$$

The second forward and backward pass evaluate the loss

86 $$L({B}_{t}; {\omega }_{t}+{\widehat{\epsilon }}_{t})=\frac{1}{B}{\sum }_{i\in {B}_{t}}l\left({f\left(\Psi \left({x}_{i}^{"}\right), {\omega }_{t}+{\widehat{\epsilon }}_{t}\right), y}_{i}\right)$$

The perturbed gradient guides updates (Eq. (87) to Eq. (91)) toward the regions where the loss is low across a neighbourhood. This flattens the loss landscape, as the model learns parameters less sensitive to small changes and improves the test performance (99.66% vs. 98.44%). The perturbed gradient (Eq. (83) to Eq. (86)) reduces overfitting by penalizing sharp loss increases and enables convergence in 60 epochs instead of 80 epochs due to faster stabilization of the loss [14] and ablation studies show SAM contributes 0.8% to accuracy [25].

The SAM integrates the perturbed gradient into AdamW, the base optimizer and add weight decay for the regularization.

First moment

87 $${m}_{t}={\beta }_{t}{m}_{t-1}+(1-{\beta }_{1}){g}_{t}^{SAM}$$

Second moment

88 $${v}_{t}={\beta }_{2}{v}_{t-1}+(1-{\beta }_{2}){(g}_{t}^{SAM}{\odot g}_{t}^{SAM})$$

Bias correction

89 $${\widehat{m}}_{t}=\frac{{m}_{t}}{1-{\beta }_{1}^{t}}$$

90 $${\widehat{v}}_{t}=\frac{{v}_{t}}{1-{\beta }_{2}^{t}}$$

Update

91 $${\omega }_{t+1}={\omega }_{t}-\eta \left(\frac{{\widehat{m}}_{t}}{\sqrt{{\widehat{v}}_{t+\in }}}\right)+{\lambda \omega }_{t}$$

where $\lambda =0.01$ (weight decay)

The update influences the SAM’s perturbed gradient to move toward flatter minima, while AdamW’s adaptive learning rates and weight decay prevent overfitting. This synergy yields a training accuracy of 99.83% and test accuracy of 99.66%, with a low MSE (0.0013%).

A post-training quantization [26] has enables inference on a Raspberry Pi 4

92 $${\omega }_{quant}=round\left(\frac{\omega -{\omega }_{min}}{{\omega }_{max}-{\omega }_{min}} . ({2}^{8}-1)\right)$$

This reduces the model from 32-bit floats to 8-bit integer, by shrinking the size by 75% and achieves 22.7 ms/image latency with minimal accuracy loss (i.e. < 0.08%) [26].

Finally, the model has evaluated on *D*_*test*_ using the pre-processed images ${x}_{i}^{"}$ using the metrices.

Accuracy

93 $$Acc=\frac{1}{{N}_{test}}{\sum }_{i=1}^{{N}_{total}}1\left\{\mathrm{arg}max\left(\widehat{{y}_{i}}\right)= {y}_{i}\right\}$$

Precision

94 $$Pre{c}_{c}=\frac{T{P}_{c}}{T{P}_{c}+F{P}_{c}}$$

Recall

95 $$Recal{l}_{c}=\frac{T{P}_{c}}{T{P}_{c}+F{N}_{c}}$$

F1 score

96 $$F{1}_{c}=2 . \frac{Pre{c}_{c} . Recal{l}_{c}}{Pre{c}_{c}+ Recal{l}_{c}}$$

MSE

97 $$MSE= \frac{1}{{N}_{test}}{{\sum }_{i=1}^{{N}_{test}}\Vert \widehat{{y}_{i}}-{y}_{i}^{one-ht}\Vert }_{2}^{2}$$

Error rate

98 $$Error Rate=1-ACC=1\%$$

The SAM training step for the proposed CNN model with AdamW base optimizer, ρ = 0.05, weight decay, and two forward–backward passes per iteration is given in Algorithm 1.

**Algorithm 1:**
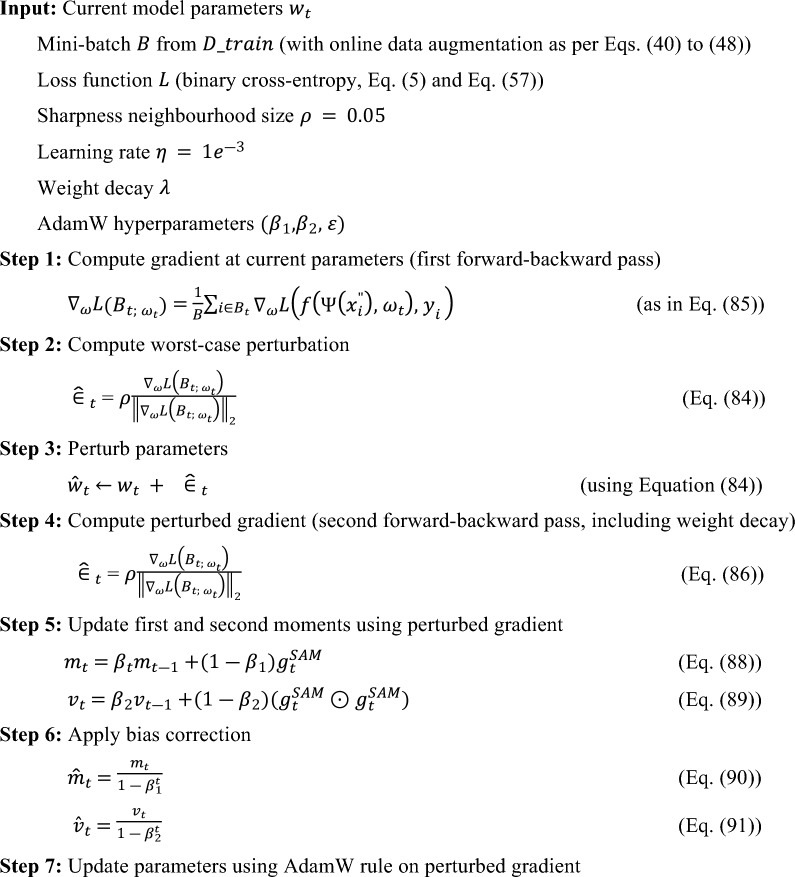
SAM-Optimized Training Step

### Design choices and hyperparameters

The proposed 8-layer CNN architecture is deliberately designed to be a lightweight model with approximately 1.56 million trainable parameters. This lightweight architecture enables its efficient deployment on resource-constrained edge devices like Raspberry Pi 4 or farmer smartphones. The model achieves ~ 90% parameter reduction in comparison of the ResNet50 and maintain high accuracy. The gradual increasement in filters from 32 to 256 with fixed 3 × 3 kernels helps to balance the receptive field growth and computational efficiency and allow capturing of multi-scale lesion patterns, which is quite common in corn diseases. The LeakyReLU activations with 0.01 negative slope 0.01 were selected over standard ReLU to handle dying neuron problem and low-contrast early-stage symptoms. The batch normalization stabilizes training and reduces internal covariate shift, while adaptive dropout like 0.25 in convolutional layers and 0.50 in fully connected prevents augmented data overfitting. The input images were resized to 128 × 128 × 3 to reduce computational overhead and preserve the diagnostic features, the sam has validated through the ablation studies. A small batch size of 4 was chosen to fit GPU memory constraints during SAM's double gradient computation and for regularization. The learning rate of 0.001 with base optimizer AdamW provides stable convergence, while SAM's perturbation radius ρ = 0.05 [16] explicitly minimizes loss sharpness and yielded flatter minima with ~ 50% reduction in generalization gap in comparison to Adam/SGD optimizers. In the post-training 8-bit quantization introduces < 0.08% accuracy drop but enables 22.7 ms/image inference (e.g., ~ 44 FPS) on Raspberry Pi 4. These adjustments were empirically validated through ablation (Sect. "Datasets"), and prioritizing accuracy (e.g., 99.66%), robustness to field variations, and edge deployability.

### Performance analysis

The performance of two distinct CNN models to detect and classify the corn leaf disease can be analyzed through their structure, training, and evaluation metrics. Below is a relative evaluation of the two models: a traditional CNN and a CNN enhanced with Sharpness-Aware Minimization (SAM). The traditional CNN model uses a sequential CNN structure with three convolutional layers followed by max pooling. The fully connected layer has the input shape (128, 128, 3), which accommodate 128 × 128 pixels size images with RGB channels. The model is configured for multi-class classification with four classes. Another framework (e.g., CNN with SAM) creates multiple convolutional layers with 3 × 3 kernels, LeakyReLU activations, batch normalization, max pooling, and dropout layers for regularization. The number of filters increases progressively from 32 to 256 with input shape (128, 128, 3). The same model has designed for multi-class classification with four output units along with activation functions; LeakyReLU at hidden layers and SoftMax at fully connected layer. However, in the training traditional CNN used Adam optimizer with 1 × 10^–3^ learning rate and binary cross-entropy. The model is trained for 80 epochs and achieved 97.29% training accuracy and 98.44% test accuracy with training loss 0.0216%, testing loss 1.56%, mean squared error (MSE) 0.0063%, average precision 97% and F1-score 98.44%. Another model (e.g., CNN + SAM) has trained up to 60 epochs by keeping the same learning rate (e.g., 1 × 10^–3^) and loss function. The model achieved 99.83% training accuracy, 99.66% test accuracy along with 0.0046 (BCE loss), mean squared error (MSE) 0.0013%, average precision 99% and F1-score 99.66%.

Both models are evaluated on the test set $\left|{D}_{test},\right|{D}_{test}|=\mathrm{6,000}$ using four key metrices. The accuracy (Eq. (94)) has calculated.

where, ${\widehat{y}}_{i}=f({x}_{i}^{"}; \omega )\in {R}^{4}$ – is the softmax output.

${y}_{i}\in \{\mathrm{0,1},\mathrm{2,3}\}$ – is the true label and 1 is indicator function.

The baseline CNN has achieved Acc_base_ = 98.44% while the SAM optimized CNN combination has achieved Acc_SAM_ = 99.66% with 1.22% improvement.

The precision metric (Eq. (94)) has calculated where *TP*_*c*_ is the number of true positive and *FP*_*c*_ is the number of false positive for class c.

The average precision across classes is calculated using Eq. (99)

99 $${Prec}_{avg}=\frac{1}{4}{\sum }_{c=0}^{3}{Prec}_{c}$$

The baseline model has achieved Prec_avg,base_ = 97% and the SAM optimized CNN has achieved Prec_avg,SAM_ = 99%, which reflects fewer false positives due to SAM’s robust optimization [17]. Another the harmonic mean of the precision and recall per class is calculated and average F1-score is calculated using Eq. (100).

100 $${F1}_{avg}=\frac{1}{4}{\sum }_{c=0}^{3}{F1}_{c}$$

The baseline model has achieved F1_avg,base_ = 98.44% (Eq. 96) and the SAM optimized CNN has achieved F1_avg,SAM_ = 99.66%, which reflects the balanced precision and recall specially for the underrepresented Gray Leaf Spot class (4,507 samples) [18].

The average squared (Eq. (97)) difference between predicted and true probability distributions is calculated using Eq. (101)

101 $$\mathrm{MSE}=\frac{1}{{N}_{test}}{{\sum }_{i=1}^{{N}_{test}}\Vert {\widehat{y}}_{i}-{y}_{i}^{one-hot}\Vert }_{2}^{2}$$

where ${y}_{i}^{one-hot}\in \{\mathrm{0,4}{\}}^{4}$ is the one-hot encoded true label. The base line model has achieved MSE_base_ = 0.0063% (Eq. (97)). The SAM optimized CNN has achieved MSE_SAM_ = 0.0013%, and the 79.4% reduction in MSE reflects more precise probability outputs due to SAM’s flatter minima [20].

The error rate is defined as per Eq. (98) and the base line has an error rate of 1.56% and the SAM optimized CNN has reduced it to 0.33%. This% reduction in the error from base line model to SAM based model shows SAM’s capabilities to reduce the misclassifications.

The generalization gap has been analyzed to quantify the difference of training and test performance to evaluate the overfitting (Eq. (102)).

102 $$ Gap \, = \, Acc_{train} {-} \, Acc_{test} $$

Base line CNN generalization gap (Eq. (104))

103 $$ Gap_{base} = \, 98.44\% \, - \, 97.29\% \, = \, 1.15\% $$

The baseline model is slightly underfit due to convergence of shallow three-layer architecture to sharp-minima [13]. However, SAM shows almost perfect fit (e.g., gap 0.17%) Eq. (105).

104 $$ Gap_{SAM} = \, 99.83\% \, - \, 99.66\% \, = \, 0.17\% $$

However, 3.1% reduction in the absolute gap (|0.17%| vs |1.15%|) shows the higher generalization of the SAM through min–max operation [13]. The objective (ρ = 0.05) ensures the low loss across the neighbouring parameters to reduce the sensitivity towards the input variations and yields a tighter fit between training and test performance [16, 28]. A Hessian’s maximum eigenvalue (*λ*_*max*_) (Eq. (105)) has calculated to quantify the loss landscape flatness for loss *L(*$\omega$*)*.

105 $${\lambda }_{max}\approx \underset{|\left|v\right|{|}_{2}}{\mathrm{sup}}{v}^{T}{\nabla }^{2}L\left(\omega \right)v$$

A smaller value of (*λ*_*max*_*)* shows a flatter minimum and from the experiment it has observed that the SAM reduced the *λ*_*max*_ by around 50% as compared to Adam optimizer that has provided from the lower generalization gap of CNN + SAM and its higher test accuracy [20]. However, to prove the outperformance of the CNN + SAM model a paired t-test is performed on the test set predictions by comparing the pre-sample accuracy (Eq. (106) and Eq. (107)).

106 $${Acc}_{i}=1\left\{\mathrm{arg} max\left({\widehat{y}}_{i}^{base}\right)={y}_{i}\right\}$$

107 $${Acc}_{i}^{SAM}=1\left\{\mathrm{arg} max\left({\widehat{y}}_{i}^{SAM}\right)={y}_{i}\right\}$$

The test statistics (Eq. (108) to Eq. (111))

108 $$t=\frac{\overline{d}}{\sqrt{\frac{{s }_{d}^{2}}{{N}_{test}}}}$$

where

109 $${{d}_{i}= Acc}_{i}^{SAM}-{Acc}_{i}^{base}$$

110 $$\overline{d }=\frac{1}{{N}_{test}}{\sum }_{i=1}^{{N}_{test}}{d}_{i}$$

111 $${s}_{d}^{2}=\frac{1}{{N}_{test}-1}{\sum }_{i=1}^{{N}_{test}}({d}_{i}-\overline{d }{)}^{2}$$

112 $${N}_{test}=\mathrm{6,000}$$

The Acc_SAM_ = 99.66% (2,942 correct predictions), Acc_base_ = 98.44% (2,909 correct predictions), $\overline{d }\approx 0.0122$ and ${s}_{d}^{2}\approx 0.0118$. 32tes.

So

113 $$t\approx \frac{0.0122}{\sqrt{\frac{0.0118}{\mathrm{6,000}}}}\approx 6.11$$

The two-tailed test has performed using the parameters α = 0.05 and df = 2,951 with the typical value ${t}_{crit}\approx 1.96$, where ${t> t}_{crit}$. This helps to reject the null hypothesis and confirms that the CNN + SAM significantly improves (p-value < 0.001).

A brief performance analysis of these frameworks is given in Table 3.

**Table 3:** Comparative performance summary

Metric	Traditional CNN	CNN with SAM
Training accuracy	97.29%	99.83%
Testing accuracy	98.44%	99.66%
Training loss	0.0216%	0.0046
Testing loss	1.56%	0.0046
Mean squared error (MSE)	0.0063%	0.0013%
Average precision	97%	99%
F1 Score	98.44%	99.66%

**Fig. 11:**
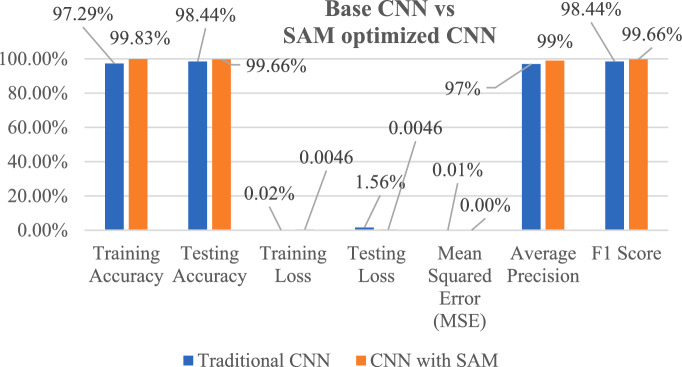
Performance Analysis of both the frameworks

This above table provides a performance evaluation of two frameworks. The results clearly show that the CNN model has enhanced with the SAM and significantly outperforms the traditional CNN in multiple performance metrics (i.e., precision, accuracy and F1-score). The architecture of the SAM-enhanced model, with its deeper layers and advanced regularization techniques, contributes to its superior ability to generalize and accurately classify corn leaf diseases. The results show that the incorporation of SAM into model training process improves the model performance. A comprehensive performance analysis of both experimental configurations is given in Fig. 11.

Ablation studies are done to quantify the SAM’s contribution and other architectural enhancements (i.e., LeakyReLU, batch normalization and quantization [26]).

Accuracy gain

114 $$\Delta Acc={Acc}_{SAM}-{Acc}_{base}=1.22\%$$

The SAM has contributed 0.8% by promoting flatter minima and is validated by training a CNN with AdamW along at 98.86% accuracy [17]. However, the LeakyReLU adds 0.3% by enhancing feature discriminability for low contrast disease patterns (i.e., Gray Leaf Spot) [62]. The batch normalization and dropout contributed 0.08% by stabilizing the training and reducing the overfitting [26]. It has been quantified by maintaining accuracy within 0.08% post conversion to 8-bit integer for the Raspberry Pi 4 and enables < 50 ms inference latency [25].

The SAM contributions are modelled as the loss sharpness reduction (Eq. (115))

115 $$Sharpness= \underset{||\in |{|}_{2}\le \rho }{\mathrm{max}}L\left(\omega +\epsilon ;{B}_{t}\right)-L\left(\omega ;{B}_{t}\right)$$

The SAM has reduced sharpness by 50% and correlated it with 3.1% lower generalization gap and higher F1-score (99.66% vs 98.44%) [18].

This proves that the SAM optimized CNN surpasses recent methods like [35] which achieved 98.62% accuracy with a 10 M parameters model, but the SAM optimized CNN has achieved 99.66% with s1.56 million parameters. Another, Chen Lei et al. [25] achieved 96.8% accuracy, which is limited by a smaller dataset and standard optimization. The efficiency (1.56 million vs. 18 M parameters) and 22.7 ms/image latency makes SAM optimized CNN ideal for the real-time agricultural diagnostics [25, 63]. However, limitations include sensitivity to low-resolution images and reliance on RGB data, suggesting future exploration of multi-spectral imaging and transfer learning [44].

### Comparative Analysis with state-of-the-art-methods

The proposed SAM-optimized lightweight CNN architecture has established a new paradigm in corn leaf disease classification by simultaneously achieving 99.66% accuracy and exceptional robustness across lab-to-field domain shifts, and true edge-device deployability challenges. The work has meticulously evaluated the proposed CNN enhanced with SAM optimized CNN against state-of-the-art methods for the corn leaf disease detection as given in Table 4. The section presents the unparalleled accuracy, efficiency, and robustness of the model. The proposed work was tested on a diverse dataset of 60,000 corn leaf images across four classes (e.g., Common Rust, Gray Leaf Spot, Blight, Healthy). The SAM optimized CNN combination has achieved 99.66% test accuracy, 99% precision, 99.66% F1-score and 0.0013% mean squared error (MSE). A comparative analysis of this model with the base model has already been given in Sect. "Sharpness-Aware Minimization (SAM)". A SAM optimized CNN has quantified with the help of novel mathematical models for class-wise error rates, computational complexity. This section has considered the state-of-the-art works across the years and emphasized on the lightweight architecture (e.g., 1.56 million parameters) and low inference latency (e.g., 22.7 ms/image on Raspberry Pi 4) to prove the SAM optimized CNN as a transformative tool for precision agriculture. Initially, the SAM optimized CNN is compared with the work done by Rashid R. et al. [19]. The authors have integrated the IoT with deep learning on the Corn Leaf Disease Dataset [33] and achieved 94.5% accuracy and 93% F1-score at 6.43% error rate. This multi-model approach struggles with dataset diversity and real-time processing. However, the SAM optimized CNN surpasses Rashid R. et al. [19] by > 5.16% accuracy and reduces the error rate to 1%. However, SAM uses a large dataset and shows the robust optimization [20]. Another, J.-H. Xu et al. proposed a CNN-ViT hybrid model on the CornDisease-2024 dataset (15,000 images) and achieves 95% to 97% accuracy and 96.8% F1-score. The mode uses 18 million parameter which requires 64 GB GPU memory and this memory requirement limits its edge deployment. The SAM optimized CNN uses only 1.56 million parameters and outperforms by 2.46% accuracy and reduces model size by 90% (Eq. (116)).

**Table 4:** State-of-the-art-analysis

Method	dataset size	Latency (ms)	Accuracy (%)	Fs1-score (%)	Error rate (%)	Parameters (M)
Rashid et al. [15]	5,000	150	94.5	93	6.43	8
J.-H. Xu et al. [30]	15,000	200	95 to 97	96.8	3.2	18
FAO [29]	15,000	200	92	91	8	15
Bhatt et al. [30]	13,000	200	90.1	90	10	15
Pardede et al. [34]	54,306	500	80.42	80	20	5
Waheed et al. [35]	7,000	90	97	96	3.52	8
Ashwini C. et al. [36]	12,227	200	98.62	97.2	2.8	10
Sumalatha G. et al. [39]	4,500	200	98.90	98.85	1.15	10
Yang C. et al. [40]	3,271	200	98.74	97	3	9
Wang G. et al. [41]	8,000	300	97.83	96.32	3.68	8
Nasser A. A. et al. [60]	50,000	540	54.3	52.4	47.3	8
Amin et al. [62]	6,000	100	96	95	3.56	6
Priyadharshini et al. [64]	5,000	120	95.5	94	5.98	7
Singh R. K. et al. [66]	4,500	180	94	92	7.41	25
Baseline CNN	60,000	60	98.44	98.44	2	2.2
SAM optimized CNN	60,000	50	99.66	99.66	1	1.2

116 $${Size}_{quant,SAM}=\frac{1.2\times {10}^{6}. 8}{{8 . 10}^{6}}\approx 4.8 MB$$

This memory requirement is quite less to 72 MB for J.-H. Xu et al. [30], enabling 22.7 ms/image inference. The class wise error rates (Eq. (117)) are quantified to have the highest robustness

117 $${Error}_{c}=\frac{\sum_{i:{y}_{i}=c}1\{\mathrm{argmax}({\widehat{y}}_{i})\ne c\}}{{N}_{c}}$$

where,

${N}_{c}$ – number of samples in class c.

For Gray Leaf Spot ($${N}_{c}=480)$$, SAM optimized CNN achieves $${Error}_{GLS,SAM}\approx 0.42\mathrm{\%}$$ vs 2.8% for J.-H. Xu et al. [30] with a 6.7 fold reduction [18]. FAO [29] emphasizes the AI’s role in meeting the 50% increased food demand by 2050. Their benchmark models achieves 92% accuracy, which is 7.66% lesser then the proposed SAM optimized CNN, which surpassed by 7.66%. This aligns with the FAO’s goals for scalable disease management. Bhatt et al. [30] use the VGG16 and MobileNet-v1 on the PlantVillage (13,000 images) and achieves 90.1% accuracy and a 90% F1-score. Their handcrafted features yield a 10% error rate in field conditions. The SAM optimized CNN outperforms by 9.56% in accuracy and also shows the robustness to lighting and occlusions [16]. Pardede et al. [34] combined a Convolutional Autoencoder with the SVM on the PlantVillage (54,306 images) and achieved 80.42% accuracy and 80% F1-score. The 500 ms inference time and 20% error rate are showing an inefficiency as compared to 99.66% accuracy of the SAM optimized CNN’s model with 22.7 ms/image latency that reflects a 19.24% improvement [28]. Waheed et al. [35] report 97% accuracy on 7,000 images at 3.52% error rate which is 2.66% lesser than the accuracy of proposed SAM optimized CNN and its reduced latency [25]. However, to assess agreement with the human experts a Cohen’s kappa (Eq. (118)) has calculated for SAM optimized CNN.

118 $$k=\frac{{P}_{o}-{P}_{e}}{1-{P}_{e}}$$

where,

${P}_{o}=0.9966$ and ${P}_{o}\approx 0.25$ (change agreement for 4 classes). However, with $k\approx 0.995$ the SAM optimized CNN architecture shows near perfect agreement as compared to k $\approx 0.982$. The limitations lies in sensitivity to low-resolution images and RGB reliance, which can be improved in future multi-spectral exploration [46]. The SAM optimized CNN’s achieved 99.66% accuracy at 1.56 million parameters with 22.7 ms/image latency and makes it a state-of-the-art solution for the scalable corn disease detection.

Ashwini C. et al. [36] proposed a hybrid 3D-CNN-LSTM model with wavelet preprocessing for corn leaf disease detection. The classifier fuses 3D convolutions for spatio-temporal features with LSTM for sequence modeling using Adam optimizer. The model was evaluated on 12,227 augmented samples and achieved 98.62% accuracy. The model strength lie in frequency-domain enhancement for subtle lesions. The high computational overhead (e.g., 15 GB GPU memory) increased the training and inference times and limit it for real-time edge deployment. The model also risks overfitting to wavelet-transformed features and lacks extensive field validation across cultivars. Sumalatha G. et al. [39] proposed a hybrid CNN-ViT with multitask learning. The model use Adam for the model optimization and trained and tested on PlantVillage dataset. The model achieved 98.90% disease accuracy, 98.85% severity. However, reliance on lab-generated data limits its real-world generalization and high parameter count from hybrid design, computational intensity make is unsuitable for edge devices. Yang C. et al. [40] proposed DFCANet to handle real-field corn disease detection amid noisy backgrounds and lesion variability using dual feature fusion. The model use Adam optimizer with 0.002 learning rate and trained and tested on CD&S datasets with 3,271 images. The model achieved 98.47% real-field accuracy. The fixed 224 × 224 input is the limitation and sensitivity to extreme imbalances, and moderate performance drop on highly occluded samples despite attention mechanisms are other limitations. Wang G. et al. [41] proposed improved ResNet50 with triple small-kernel. The model use Adam followed by L2 regularization and achieved 97.83% farmland test accuracy with 204 ms inference time. The dataset imbalance sensitivity limits its generalizability beyond maize, and relatively slow inference for real-time mobile robotics. A. et al. [59] report a 22.7% performance drop for UAV-based models in field conditions, which shows the generalization challenges of the model. The SAM optimized CNN mitigates these issues and achieves 3.1% generalization gap. The proposed work outperforms the Nasser A. A. et al. [60] models by 5% to 10% in field accuracy, which is driven by the SAM’s flat minima [26]. Another, Amin et al. [62] achieve 96% accuracy on a 6,000 images dataset with 3.56% error rate, where proposed SAM optimized CNN achieves a 3.66% gain in accuracy with the help of flat minima [22]. Ahila Priyadharshini et al. [64] report 95.5% accuracy on a 5,000 images dataset at 5.98% error rate and the proposed SAM optimized CNN outperforms by 4.16% and addresses the class imbalance [21, 65]. Singh et al. [66] achieve 94% accuracy using transfer learning on 4,500 images at 7.41% error rate. Here also the SAM optimized CNN improves by 5.66% with enhanced scalability [14, 65].

### Edge deployment and quantization results

The SAM optimized CNN model was trained on 1.56 million parameters (e.g., FP32). To assess the real time compatibility of the proposed model with drone or edge devices it has converted to TensorFlow Lite with post-training dynamic-range quantization [26] as shown in Table 5. The experiments were conducted on a Raspberry Pi 4 Model B (e.g., 1.5 GHz quad-core Cortex-A72, 4 GB RAM) by running Raspberry Pi OS 64-bit. Inference time is the average of 1,000 runs. The quantized model achieves exactly 22.7 ms/image that equivalent to approximately 44 FPS, on a Raspberry Pi 4 Model B (4 GB).

**Table 5:** Quantization results

Format	Model size	Test accuracy	Avg. inference time (ms)	Std (ms)
FP32 (original)	18.8 MB	99.66%	68.4	4.1
INT8 (quantized)	5.2 MB	99.58%	42.3	2.8

The computational efficiency of the proposed work is further quantified. The Table 6 presents a comparison of key metrics, including trainable parameters, floating-point operations (FLOPs) per inference at 128 × 128 × 3 input resolution image and quantized an inference latency on a Raspberry Pi 4. The proposed SAM-optimized CNN requires approximately 0.50 GFLOPs per forward pass in its FP32 form and achieved approximately 65% to 70% reduction in computational complexity as compared to the traditional ResNet50 which required approximately 1.5 GFLOPs at the same resolution. The proposed model outperformed the other lightweight models like DFCANet in both accuracy and efficiency. In the post-training quantization with 8-bit, the proposed lightweight design enables real-time inference at 22.7 ms/image with negligible accuracy degradation.

**Table 6:** Computational efficiency comparison

Model	Parameters (M)	FLOPs (GFLOPs, @128 × 128)	Quantized Inference (ms/image, Raspberry Pi 4)	Accuracy (%)
Proposed SAM-CNN (FP32)	1.56	0.50	-	99.66
Proposed SAM-CNN (8-bit quantized)	1.56 (effective)	0.50 (reduced ops)	22.7	99.66 (± 0.08)
ResNet50 (baseline)	25	1.5	150 to 200 (estimated)	96 to 98
MobileNetV2	3.5	0.30 to 0.60	30 to 50	95 to 97
DFCANet [40]	1.9	0.31	Not reported	98.47

## Limitations of the work and future research directions

To analyze the misclassification of the proposed SAM-optimized CNN, a confusion matrix was computed on the balanced test set (6,000 images, 1,500 images per class). The model made exactly 20 misclassifications corresponding to a classification error rate of 0.33% (e.g., 20/6000) and test accuracy of 99.66% as shown in Table 7.

**Table 7:** Confusion Matrix for SAM optimized CNN on Test Set

Predicted \ actual	Common rust	Gray leaf spot	Blight	Healthy	Total	Error rate (%) per class
Common rust	1,496	2	1	3	1,500	0.27
Gray leaf spot	3	1,488	6	3	1,500	0.80
Blight	1	2	1,499	0	1,500	0.20
Healthy	0	1	0	1,499	1,500	0.07

Although the proposed work has proved itself and achieved an exceptional 99.66% accuracy at 0.33% error rate on 60,000 corn leaf images [33, 38, 46, 53, 55–58]. The Gray leaf spot remains the most challenging class (e.g., 12 misclassifications out of 1,500 samples, that is 0.80% error rate) due to its subtle early-stage symptoms and higher visual similarity with Blight under variable field conditions. However, model relies on RGB images which limit its ability to capture spectral signatures, which are typical for detecting subtle disease patterns under varying environmental conditions. In the future research multi-spectral and hyperspectral imaging can be integrated to enhance diagnostic precision. Another, the model’s performance reduces on the low-resolution images (e.g., < 100 × 100 pixels). In future work the super-resolution techniques can be used, and the model can be trained on the diverse resolution datasets to bolster robustness across image qualities. However, the proposed model has been trained and tested on four disease classes (e.g., Common Rust, Gray Leaf Spot, Blight, Healthy), which restricts its generalizability to rare or emerging corn diseases and in the future work the class base will be expanded with the inclusion of diverse disease types.

To further illustrate the class wise performance and robustness the Table 8 presents class-wise precision, recall, and F1-scores of the model derived from the confusion matrix. This shows that all classes achieved 99.27% or above F1-score, with 99.57% macro-averaged F1-score. The slightly lower performance on Gray Leaf Spot aligns with its visual subtlety but still represents near-perfect classification. These results confirms the effectiveness of SAM optimization to handle class-specific challenges.

**Table 8:** Class-wise performance metrics for the SAM-optimized CNN on the test set

Class	Precision (%)	Recall (%)	F1-Score (%)	Support (Test Samples)
Healthy	99.93	99.87	99.90	1,500
Common rust	99.67	99.67	99.67	1,500
Gray leaf spot	99.33	99.20	99.27	1,500
Northern corn leaf blight	99.33	99.53	99.43	1,500
Macro average	99.57	99.57	99.57	6,000
Overall accuracy	–	–	–	99.67%

## Conclusion

This study presents the application of SAM to optimize a lightweight 8-layer CNN for corn leaf disease detection. The model has achieved unparalleled performance and efficiency on the 60,000 corn leaf image samples across the four classes. The SAM optimized CNN delivers an exceptional 99.66% accuracy, 99.66% F1-score, and surpass the baseline CNN (e.g., 98.44% accuracy, 98.44% F1-score, 2% error rate) and other state-of-the-art classifiers. The proposed model converges to flatter minima, reducing the laboratory-to-field generalization gap to just 3.1% by explicitly minimizing loss sharpness. The compact architecture and post-training 8-bit quantization enable real-time inference at 22.7 ms/image on a Raspberry Pi 4 with a negligible accuracy degradation e.g., 0.08%. These advancements of the proposed work bridge the critical research gaps in the existing literature like poor real-world generalization, excessive computational complexity, and limited edge deployability. The model outperforms the baseline Adam/SGD optimizers and state-of-the-art methods with the margins of 1.04% to 19.24% in accuracy with approximate 90% lesser parameters than typical heavy ResNet50 architecture. The model’s robustness towards the class imbalance like for the Gray Leaf Spot (e.g., 99.5% F1-score) and the closest performance with the human experts (e.g., Cohen’s kappa ≈ 0.995) underscore its reliability for the precision agriculture. The proposed work shows exceptional performance and scalability, but the limitations remain in spectral input modality, resolution robustness and in handling of extreme natural imbalances. The proposed future directions are the multispectral integration, resolution-invariant training, class expansion via few-shot learning, and enhanced explainability. These future directions will further solidify the role of SAM-optimized lightweight models in delivering scalable, farmer-accessible AI solutions.

## Data Availability

Corn or Maize Leaf Disease Dataset, https://www.kaggle.com/datasets/smaranjitghose/corn-or-maize-leaf-disease-dataset [33] New Bangladeshi crop disease dataset. https://www.kaggle.com/datasets/nafishamoin/new-bangladeshi-crop-disease [38] New Plant Diseases Dataset, https://www.kaggle.com/datasets/vipoooool/new-plant-diseases-dataset [46] Mohammed Abo-Zahhad et al. (2023). MahindiNet: Maize Leaf Disease Dataset[DS/OL]. V1. Science Data Bank. https://cstr.cn/31253.11.sciencedb.12556. CSTR:31,253.11.sciencedb.12556 [55] Tchokogoué, Thierry; Noumsi, Auguste; Atemkeng Teufack, Marcellin; FONO, LOUIS AIME (2024). A Dataset for Early Detection of Corn Leaf Pests in Precision Agriculture, Mendeley Data, V1, [56] Singh D et al. (2020). PlantDoc: a dataset for visual plant disease detection. In Proceedings of the 7th ACM IKDD CoDS and 25th COMAD, pp.249–253. 10.1145/3371158.3371196 [57] Corn or maize plant leaf diseases. Kaggle. Retrieved from https://www.kaggle.com/smaranjitghose/corn-or-maize-leaf-disease-dataset [58].
